# A Real Time PCR Platform for the Simultaneous Quantification of Total and Extrachromosomal HIV DNA Forms in Blood of HIV-1 Infected Patients

**DOI:** 10.1371/journal.pone.0111919

**Published:** 2014-11-03

**Authors:** Anna Casabianca, Chiara Orlandi, Benedetta Canovari, Maddalena Scotti, Marcello Acetoso, Massimo Valentini, Enzo Petrelli, Mauro Magnani

**Affiliations:** 1 Department of Biomolecular Sciences, University of Urbino “Carlo Bo”, Urbino (PU), Italy; 2 Azienda Ospedaliera Ospedali Riuniti Marche Nord - Presidio Ospedaliero San Salvatore, Pesaro (PU), Italy; George Mason University, United States of America

## Abstract

**Background:**

The quantitative measurement of various HIV-1 DNA forms including total, unintegrated and integrated provirus play an increasingly important role in HIV-1 infection monitoring and treatment-related research. We report the development and validation of a SYBR Green real time PCR (*TotUFsys* platform) for the simultaneous quantification of total and extrachromosomal HIV-1 DNA forms in patients. This innovative technique makes it possible to obtain both measurements in a single PCR run starting from frozen blood employing the same primers and standard curve. Moreover, due to identical amplification efficiency, it allows indirect estimation of integrated level. To specifically detect 2-LTR a qPCR method was also developed.

**Methodology/Findings:**

Primers used for total HIV-1 DNA quantification spanning a highly conserved region were selected and found to detect all HIV-1 clades of group M and the unintegrated forms of the same. A total of 195 samples from HIV-1 patients in a wide range of clinical conditions were analyzed with a 100% success rate, even in patients with suppressed plasma viremia, regardless of CD4+ or therapy. No significant correlation was observed between the two current prognostic markers, CD4+ and plasma viremia, while a moderate or high inverse correlation was found between CD4+ and total HIV DNA, with strong values for unintegrated HIV DNA.

**Conclusions/Significance:**

Taken together, the results support the use of HIV DNA as another tool, in addition to traditional assays, which can be used to estimate the state of viral infection, the risk of disease progression and to monitor the effects of ART. The *TotUFsys* platform allowed us to obtain a final result, expressed as the total and unintegrated HIV DNA copy number per microgram of DNA or 10^4^ CD4+, for 12 patients within two working days.

## Introduction

HIV infection has been transformed over the past two decades from a lethal disease to a manageable chronic condition thanks to the advent of combination antiretroviral therapy (ART). Nevertheless, virus persistence in reservoirs prevents complete virus eradication in patients treated with current therapies [1]–[3]. In recent years, *a)* the introduction of new drugs (e.g. viral integrase inhibitors, co-receptor antagonists), in addition to the classic inhibitors of reverse transcriptase and protease, which interfere with other steps in the virus life cycle, and/or new therapeutic vaccinations, *b)* efforts to gain a greater understanding of the nature and role of the reservoir in AIDS pathogenesis and *c)* low-level persistent viremia despite clinically successful antiretroviral therapy have encouraged a careful analysis of the kinetics and relative contributions of the viral DNA to HIV-1 replication and latency during disease progression and ART treatment.

Total cell-associated HIV-1 DNA (total HIV DNA) is present in infected cells in three major forms that reflect the different stages and fates of development during viral replication: integrated proviral DNA (IDNA) and unintegrated (extrachromosomal) forms (UF) including both linear and circular DNA (1-LTR and 2-LTR). Several authors have shown the presence of small amounts (1% or more) of the aberrant circular forms. HIV-1 infection *in vitro* and *in vivo* results in an abundance of UF, regardless of cell type and activation status [4]–[7]. Blood, lymphoid tissue and brain tissue show a ratio of extrachromosomal to integrated forms of 99∶1, while the ratio linear/1-LTR/2-LTR is 20∶9∶1 [1], [8], [9]. Regarding stability, the following order was found: integrated DNA>circular DNA (1-LTR and 2-LTR)>linear DNA. The detection of high levels of unintegrated DNA in the brain has been associated with the development of AIDS dementia [9]. In particular, 2-LTR circles, have been suggested as a possible marker of recent infection due to their labile nature, although stable unintegrated forms have been shown to exist, and hence their utility as a clinical marker of recent infection is questionable. 2-LTR circles are often viewed as overall markers of all unintegrated forms, although they are present at relatively low levels compared to other HIV DNA species. The extrachromosomal forms are biologically active: they produce functional viral proteins, are toxic to the cell and can trigger the apoptotic cascade [7], [10]–[12].

Currently, HIV-1 RNA levels and CD4+ T lymphocyte counts are the standard markers used in clinical practice for the management and the monitoring of HIV-1 infected patients. CD4+ T cell counts yield information on the patient's immunological status and the HIV-RNA load provides information on the extent of viral replication at the time of the assay. At present, antiretroviral protocols use drugs that suppress the replicative ability of HIV-1 to the point that circulating virus in plasma becomes undetectable using the standard commercial viral RNA detection assays (20–50 copies/ml). However, low levels of free virus can still be detected in a majority of patients on ART using ultrasensitive assays. After several years of therapy, this residual viremia reaches a plateau of 1–10 copies/ml and does not appear to decline any further [13]. Hence, in this scenario, it would be useful to have additional virological markers for monitoring and predicting therapy responses and for measuring the degree of HIV-1 persistence in patients on ART. Assays that quantify viral DNA have been already developed and are taking an important role in HIV cure-related research. Total HIV DNA has been used for a number of years and is currently the most feasible tool available for large-scale clinical trials and cohort studies [14]–[20]. Several reports have investigated the prognostic value of HIV DNA measurement as a marker of disease progression and treatment efficacy [21]–[27]. HIV DNA provides essential information on the reservoir and dynamics of the HIV-1 infection, especially in patients with undetectable plasma viremia, in whom HIV DNA could represent the only biomarker of viral activity that can be easily detected. 

**Figure 1:**
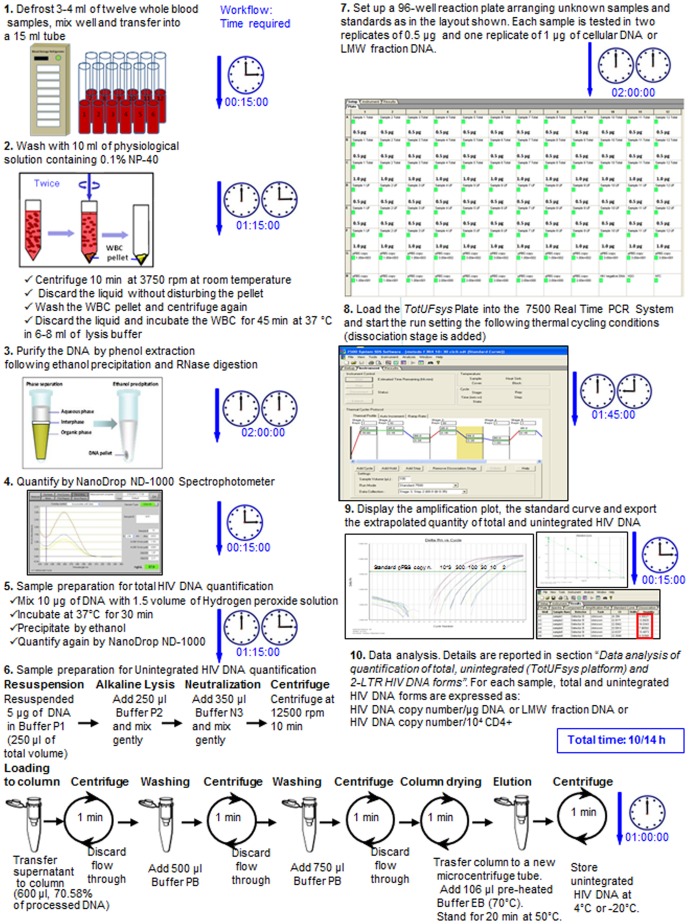
Complete workflow of the whole procedure for the quantification of total and unintegrated HIV DNA forms in 12 samples, from frozen blood until data analysis.

The aim of this work was to evaluate the reliability and usefulness of the simultaneous quantification of total and all unintegrated HIV DNA forms (linear+1- and 2-LTR circles) in a wide range of clinical situations. We used a high performance workflow and a PCR plate layout (*TotUFsys* platform), starting from a single cellular DNA recovered once from whole blood of HIV-1 infected patients. These patients reported to the reference hospital for routine clinical tests. Based on a previously developed strategy [28], we improved the whole blood leukocyte assay (pbs-rtPCR) in terms of robustness for total HIV DNA quantification and also developed a new SYBR Green qPCR, which was optimized and validated for quantifying all unintegrated forms. For a further comprehensive analysis of the clinical samples, we also developed a SYBR Green qPCR based method to specifically detect 2-LTR circles in the same cellular DNA samples used for the quantification of total and unintegrated HIV DNA. Both the *TotUFsys* platform and the 2-LTR assay were developed analyzing the Guidelines for the Validation of Analytical Procedures (http://www.ich.org). An appropriate exogenous control was added to monitor the various steps of the procedure, and negative controls were also tested. The complete workflow of the whole procedure for the quantification of total and unintegrated HIV DNA forms is illustrated in Figure 1.

## Results

### Patient characteristics

Clinical characteristics of patients and their different clinical pictures are summarized in Table 1.

**Table 1:** Clinical characteristics of the patients.

Characteristic	Value
Male	39 (66%)
Female	20 (34%)
Age, median years (range)	44 (27–70)
CD4+ T cell count at nadir, median cells/µl (range)	250 (2–1200)
HIV-1 RNA load at nadir, median copies/ml (range)	10∧5 (<50-10∧6)
Years since diagnosis, median years (range)	4 (<1–28)
Treatment-naïve	11 (19%), 32 blood samples
ART-experienced	48 (81%), 163 blood samples
Years on ART, median years (range), n = 48	2.5 (<1–19)
Multidrug resistant HIV-1 infection (MDR)	21 (36%), 85 blood samples
HCV, HBV, Syphilis, TBC co-infected	23 (39%)

### Primer and diagnostic specificity

**Figure 2:**
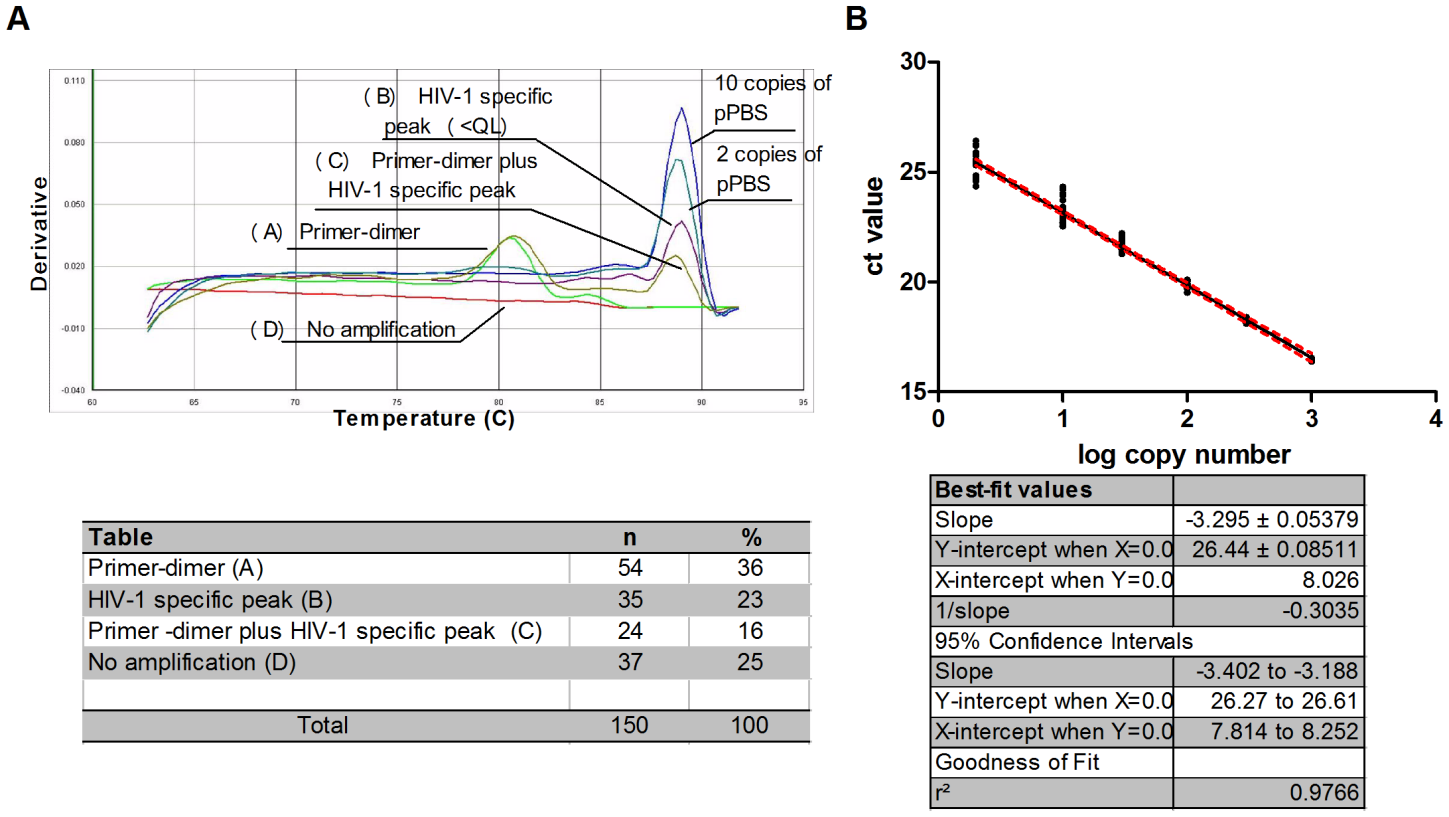
Post-PCR melt curve analysis and standard curve. (A) Different dissociation curves that are shown in percentages of their relative amounts in the analysis of 150 HIV-1 negative DNA samples. Only 10 and 2 copies of standard are displayed. (B) Mean standard curve obtained in the *TotUFsys* PCR experiments (n = 8).

Primer specificity for HIV-1 clades in group M was confirmed *in silico* by BLAST (Figure S1) [29], and also by real time PCR using different HIV-1 subtypes and HIV-2 ROD complete proviral sequences [28]. No cross-reactivity with retroviral endogenous sequences (HERV) [30] was detected in 100 HIV-1 negative blood donors using real time PCR [28]. Moreover, an additional 150 HIV-1 negative samples were checked. Samples showing only a very weak peak, consistently below 2 copies (Ct mean value±SD: 26.60±0.87, n = 35), were considered as nonspecific PCR signals (Figure 2, panel A and Table S1).

### Standard curve, sensitivity and reproducibility of the assay (*TotUFsys* platform)

For HIV DNA quantification, the pPBS standard curve was constructed with half-log plasmid serial dilutions from 10∧3 to 10 copies and 2 copies. The quantification limit (QL) was set at 2 copies/PCR reaction. The standard was analyzed in the presence or absence of 0.5 µg of HIV-1 negative DNA (background DNA, bk DNA). The two curves revealed no differences in their linear range of quantification or PCR efficiency (Δslope<0.1, Applied Biosystems User Bulletin No. 2 http://www3.appliedbiosystems.com/cms/groups/mcb_support/documents/generaldocuments/cms_040980.pdf, 1997). Moreover, in the presence of 0.5 µg of bk DNA no inhibitory effect was detected (2-copy Ct value: 25.47±0.89 and 25.35±0.59 with or no bk DNA, respectively, Table S2). Hence, the standard without bk DNA was used for total and unintegrated HIV DNA measurements (Figure 2, panel B and Table S3). The method's reproducibility was calculated by the percentage of coefficient variation for Ct values (CV_Ct_) and for copy numbers (CV_Cn_). The CV_Ct_ and CV_Cn_ mean values of pPBSstd were 1.32% and 20%, respectively (Table S4). These data were confirmed assaying the reproducibility of some samples as inter and intra-assay variations (Table S5).

### Unintegrated HIV DNA separation from high molecular weight DNA

The selective separation of extrachromosomal HIV DNA from high molecular weight (HMW) genomic DNA, which could harbor integrated HIV-1 provirus, was performed adapting a plasmid DNA purification through column chromatography on silica gel procedure. This strategy was reported for the first time by Sharkey *et al.* [6] for the selective detection of 2-LTR circles and by others [15], [31] using a PBMC pellet of about ∼2–6×10∧7 cells. In the present investigation, the possible use of a small amount of cellular DNA (5 µg) was evaluated. This DNA was obtained by the phenol extraction procedure able to ensure an HMW of typically ∼100–200 Kb [32] and non degraded DNA. The genomic DNA extracted using commercial kits (<50 kb) could be co-purified in the eluate fraction. To assess the performance of this approach, 5 µg of an HMW DNA sample and an equal amount of the molecular weight Marker II (Lambda DNA/HindIII), used as a procedure monitor, were processed in columns and the eluate fractions were analyzed by agarose gel electrophoresis. The results showed the absence of HMW DNA>10 Kb and a slight degradation of the DNA sample, demonstrating the ability of the procedure to selectively recover the DNA which approximates the genome length of HIV-1 (Figure S2). To assess the extent of DNA degradation, the β-actin housekeeping gene was amplified and quantified on ACTstd. The percentage was estimated at 5.0±1.3% (mean±SD, n = 20), revealing the presence of a negligible amount of HMW DNA fragments co-purified in the low molecular weight (LMW) DNA fraction, containing unintegrated HIV DNA (Table S6). Clearly, this drawback was intrinsic in the spin-column method itself, and did not affect the final results. To measure the actual recovery of unintegrated forms, the 13 Kb pEXg plasmid, which simulates the HIV-1 genome length, was included at the beginning of the column separation procedure as an exogenous standard. A typical experiment involved the addition of known amounts of the pEXg (10∧2 or 10∧3 copies) to 5 µg of cellular DNA, followed by the evaluation of the recovered quantity by qPCR. Repeated experiments showed high levels of recovery (94%–98%, Table S7). We used 10∧2 and 10∧3 plasmid copies because the expected copies/PCR were 10 and 100 respectively, reflecting the HIV DNA content found in HIV-1 positive patients (range from 2 to 108 in 73 samples) [28].

### Quantification of total and unintegrated HIV DNA forms in blood samples

In order to confirm that the *TotUFsys* was able to detect and quantify the different HIV DNA forms in a range of clinical pictures, a total of 195 HIV-1 positive blood samples were tested. The samples were collected from ART-experienced subjects (163, 84%) and from treatment-naïve patients (32, 16%). To enhance precision and sensitivity, HIV DNA copy numbers were measured in a replicate of 0.5–1.0 µg of DNA or LMW fraction DNA from 2 to 8 and normalized to 1 µg of cellular DNA. Table 2 shows the quantifications of the total and unintegrated HIV DNA of 5 representative samples and how the final data were calculated and normalized.

**Table 2:** Quantification of total and unintegrated (UF) HIV DNA copy number in five different samples.

Run	Sample 1		Sample 2		Sample 3		Sample 4		Sample 5	
	Total HIV DNA	UF HIV DNA	Total HIV DNA	UF HIV DNA	Total HIV DNA	UF HIV DNA	Total HIV DNA	UF HIV DNA	Total HIV DNA	UF HIV DNA
1^st^ qPCR	15	5	14	5	19	7	21	16	39	17
1^st^ qPCR	14	2	10	3	24	10	22	12	50	17
2^nd^ qPCR	11	2	11	5	24	10	28	18	43	22
2^nd^ qPCR	12	3	16	5	20	11	24	13	46	16
2^nd^ qPCR	13	6	13	5	23	12	20	11	50	22
2^nd^ qPCR	11	4	12	8	24	7	23	14	45	20
2^nd^ qPCR	20	3	17	5	22	11	25	12	41	20
2^nd^ qPCR	15	3	10	4	21	12	25	16	43	17
Coefficient of variation	21.25%	40.41%	20.52%	28.28%	8.86%	20.00%	10.91%	17.50%	8.88%	12.80%
Lower 95% CI of mean	11	2	11	4	20	8	21	12	41	17
Upper 95% CI of mean	16	5	15	6	24	12	26	16	48	21
Sum	110	29	101	39	176	81	189	113	358	149
aCopies/µg	28	7	25	10	44	20	47	28	89	37
bCopies/ml of blood	780	195	1309	524	2667	1212	1846	1100	4124	1715
cCopies/10^4^ CD4+	13	3	55	22	24	11	48	29	104	43

a The results were obtained dividing the sum of the copy number from a total of eight replicates (two 0.5 µg replicates in the 1^st^ qPCR and six 0.5 µg replicates in the 2^nd^ qPCR) by 4 and expressed as HIV DNA copy number/µg of DNA.

b Taking into account the WBC number, the results can be reported as copies/ml of blood with the formula: (copies/µg DNA)×WBC no. per ml/142857 cells, assuming that 142857 cells are present in one µg of DNA. WBC counts/µl are 3980 (sample 1), 7480 (sample 2), 8660 (sample 3), 5610 (sample 4), and 6620 (sample 5).

c Taking into account %CD4+, the results can be reported as copies/10^4^ CD4+ with the formula: [(copies/µg DNA)/(CD4+/WBC×142857 WBC)]×10^4^. The %CD4+ are: 15.0% (sample 1), 3.2% (sample 2), 12.9% (sample 3), 6.8% (sample 4), and 6.0% (sample 5).

**Figure 3:**
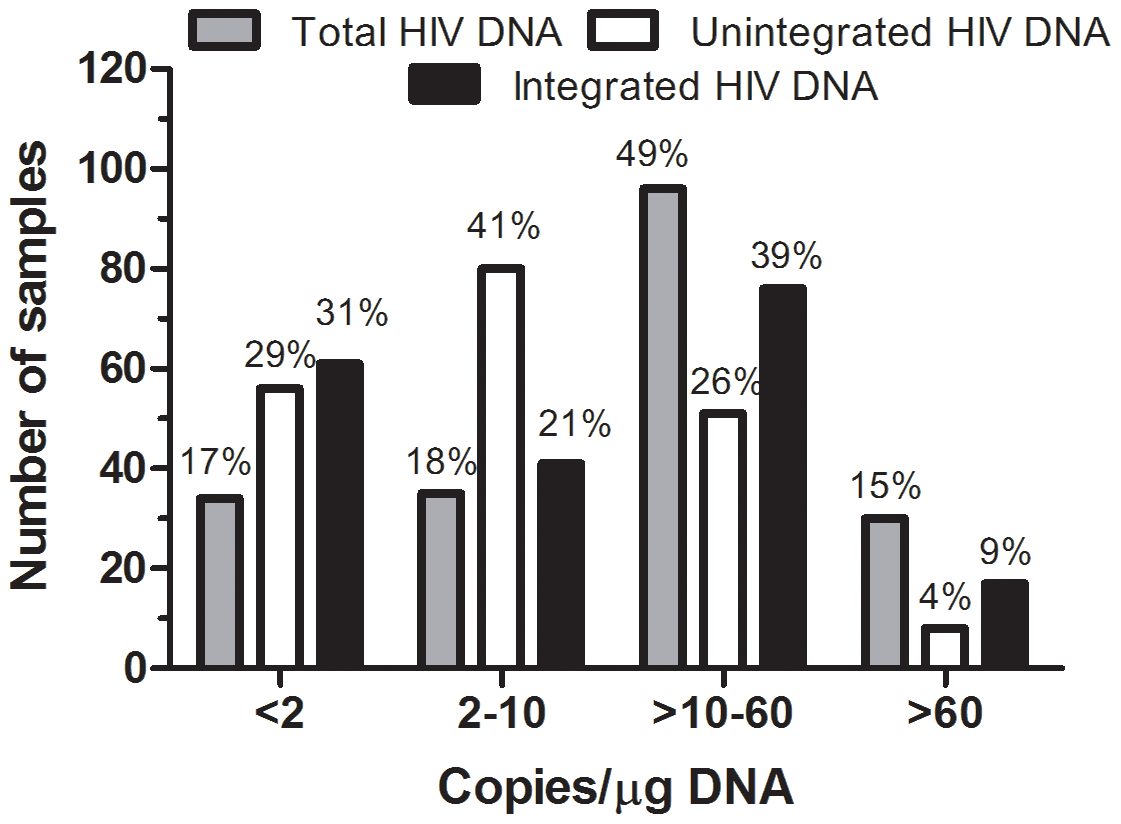
Distribution of total, unintegrated and integrated HIV DNA copy number in four range: very low (<2 copies), low (2–10), medium (>10–60) and high (<60) for 195 blood samples.

For 100% of the analyzed samples we were able to give a final result as the total, unintegrated and integrated provirus HIV DNA copy number, regardless of plasma viremia, CD4+ T cell count or therapy (Table S8). Provirus was obtained by subtracting unintegrated from total HIV DNA. For total HIV DNA, the highest percentage (95%) of quantified samples (QL, 2 copies) was found in samples with a CD4+ T cell count <350, whereas the lowest percentage (81%) was found in samples with plasma viremia >50 copies/ml and in ART–naïve samples, although it should also be noted that these two groups were smaller than the other groups. For unintegrated HIV DNA, the highest value was found in samples with plasma viremia >50 copies/ml and in ART–naïve samples (75%). Of note, although the differences are small, there were more samples with undetectable integrated HIV DNA in patients under treatment, with suppressed plasma viremia and higher CD4+ T cell counts. The median total HIV DNA was 17 (range: <2–923, IQR: 5–40) and 693 copies (range: 12–30044, IQR: 180–1584) per µg of DNA or ml of whole blood, respectively; the median unintegrated HIV DNA was 5 (range: <2–238, IQR: <2–14) and 192 copies (range: 12–6931, IQR: 76–549). The integrated HIV DNA revealed a median of 9 (range: <2–802, IQR: <2–26) and 355 (range: 12–26105, IQR: 72–1016) per µg of DNA or ml of whole blood, respectively. In Figure 3 are reported the percentages of copy number distribution of the total, unintegrated and integrated HIV DNA forms. We evidenced that 65% of sample quantifications of the various forms were in the range 2–60 copies, supporting the use of the half-log dilution standard curve to enhance precision of the data. To account for the variation in the number of CD4+ T cells in different samples, the results were further normalized by the CD4+ percentage of total WBC and expressed as copies/10^4^ CD4+. This normalization is based on the assumption that most of the HIV DNA is in the CD4+ T cells [33], [34] and on our statistical analysis which showed an average of 10867 CD4+ analyzed per µg of DNA (median, range: 8632, 158–34869). Median HIV DNA was 21 (range: <2–2407, IQR: 6–50) and 6 (range: 1–1457, IQR: 2–17) copies per 10^4^ CD4+ for total and unintegrated forms, respectively. The integrated form showed a median of 10 (range: 0–950, IQR: 3–32) copies per 10^4^ CD4+. Table 3 shows the differences in HIV DNA load in some samples according to the normalization procedure that was chosen. Individuals with quite similar data trends regarding total HIV DNA copies/µg (or ml of blood) recorded in two sequential visits (patients 9–35), actually show at least a two-fold decrease in the content of HIV DNA copies/µg or even a nearly 20-fold decrease, considering the same data expressed for 10^4^ CD4+ T cells. This decrease correlates with the increase in equal measure of the percentage of CD4+ T cells. The decrease in HIV DNA content is actually much more evident considering the data normalized for 10^4^ CD4+, a nearly five-fold decrease (patients 41, 26, 22). Likewise, an apparent two- to five-fold increase (patients 33, 52, 56) results in no change in the HIV DNA load for 10^4^ CD4+. Due to the impact of the normalization procedure on the quantification of HIV DNA, we decided henceforth to conduct each type of subsequent analyses comparing the data obtained by qPCR (copy number/µg DNA) to those expressed for 10^4^ CD4+ T cells, considering these data to be more informative than HIV DNA per ml of blood.

**Table 3:** The effect of the data normalization procedure on the quantification of HIV DNA in blood samples from HIV-1 positive subjects.

Patient	Time point	WBC cell count/µl	CD4+ T cell count/µl	% CD4+	Fold change	Total HIV DNA copy no.					
						per µg of DNA	Fold change	per ml of blood	Fold change	per 10^4^ CD4+	Fold change
9	1	5100	340	6.7	2.6	106	1.1	3784	1.2	111	2.4
	4	3940	683	17.3		115		3172		46	
10	1	4890	110	2.2	2.4	62	1.1	2122	1.6	193	2.2
	7	7130	389	5.5		68		3394		87	
16	1	6890	326	4.7	1.8	15	1.0	723	1.7	22	1.8
	9	4160	354	8.5		15		437		12	
18	1	5200	470	9.0	2.2	63	0.9	2293	1.1	49	2.3
	2	6030	1190	19.7		59		2490		21	
19	1	7690	100	1.3	1.8	39	1.0	2099	1.2	210	1.9
	2	6320	150	2.4		38		1681		112	
30	1	8970	40	0.4	6.9	18	0.9	1130	1.1	283	7.4
	6	8490	263	3.1		17		1010		38	
35	1	1810	2	0.1	17.0	38	0.9	481	2.8	2407	18.0
	2	5420	102	1.9		36		1366		134	
41	1	2370	42	1.8	4.4	10	2.0	166	1.1	40	10.0
	3	5130	400	7.8		5		180		4	
26	1	12100	40	0.3	4.3	21	5.3	1779	15.6	445	22.3
	5	4060	58	1.4		4		114		20	
22	1	4880	120	2.5	4.5	170	7.4	5807	8.3	484	34.6
	6	4340	477	11.0		23		699		14	
33	1	7400	157	2.1	2.0	14	1.9	725	1.7	46	1.0
	4	6390	273	4.3		27		1208		44	
52	1	3210	45	1.4	3.5	23	3.0	517	3.1	115	0.8
	2	3300	163	4.9		69		1594		97	
56	1	9540	70	0.7	6.9	20	5.4	1336	2.5	186	0.8
	3	4500	227	5.0		107		3371		148	

### Correlations between study parameters in blood samples

**Figure 4:**
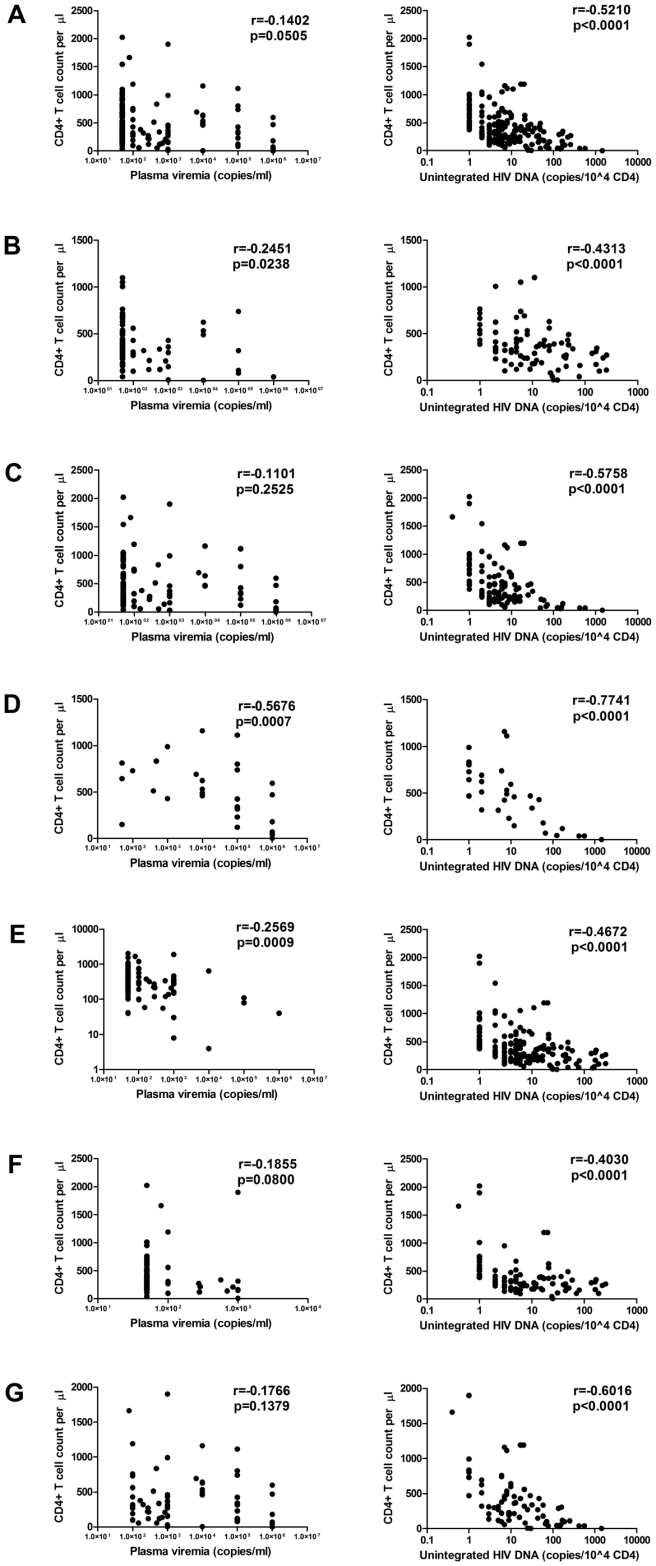
Correlations between study parameters in blood samples. Correlations between plasma viremia and CD4+ T cell counts (left panel) and between unintegrated HIV DNA and CD4+ T cell counts (right panel) in (A) a total of 195 blood samples, (B) 85 blood samples collected from patients with multidrug-resistant HIV-1 infection, (C) 110 blood samples collected from patients who had no evidence of resistance, (D) 32 treatment-naïve samples, (E) 163 ART samples, (F) 90 under RAL samples and (G) 72 samples with HIV-1 RNA loads above 50 copies/ml of plasma. Only the correlation between CD4+ and unintegrated HIV DNA is shown because it is stronger than the correlation between CD4+ and total HIV DNA.

The correlations between the amount of HIV DNA and plasma viremia or CD4+ T cell counts and between HIV-1 RNA and CD4+ were examined using Spearman's rank test. Most correlations were found when the data were expressed for 10^4^ CD4+ (Table S9). When all 195 samples were analyzed together, no significant correlation was observed between plasma viremia and CD4+ (r = −0.14, p = 0.05) and there was a marginal positive correlation between plasma viremia and the amount of unintegrated HIV DNA (r = 0.31, p<0.0001). However, there was a moderate inverse correlation between CD4+ T cell counts and both total (r = −0.48, p<0.0001) and UF HIV DNA (r = −0.52, p<0.0001). Due to the wide range of clinical situations within our cohort of samples, correlations were evaluated in different subsets, dividing them into six groups according to various criteria. Two groups were defined according to evidence of resistance: MDR (Multidrug resistant HIV-1 infection) and non-MDR. Three groups were identified on the basis of therapy: ART, under RAL, and without therapy (Naïve). Finally, a sixth group was defined according to measurable plasma viremia (HIV-1 RNA>50 copies/ml). There was an inverse moderate correlation between viral load and CD4+ T cell counts only in the treatment-naïve group (r = −0.57, p<0.005). Plasma viremia showed a weak positive correlation with HIV DNA (total and UF) in the non-MDR group (r≥0.31, p<0.005), it correlated strongly with HIV DNA in the treatment-naïve group (r≥0.66, p<0.0001) and in the samples with measurable plasma viremia (r≥0.40, p≤0.005). Each of these correlations was stronger when the UF were considered. Interestingly, there was consistently a significant inverse correlation between CD4+ and HIV DNA in all the groups examined (r within −0.40 and −0.77, p<0.0001). Such inverse correlations were stronger for UF (Figure 4). We selected 45 subjects for whom at least two sequential samples were available, to compare samples from an arbitrary time zero (START, first sampling available) to those taken at the end of the observation period (STOP, last sampling available) and the following groups were analyzed: treatment-naïve, under ART, ART-subjects under RAL intensification and a last group was formed by combining the latter two groups (Table S10). It should be noted that for the 45 patients, while a modest correlation between plasma viremia and CD4+ (r = −0.29, p = 0.06) or HIV DNA (r≥0.40, p≥0.01) is reduced to insignificant values from the beginning to the end of the observation period, the higher inverse correlation between CD4+ and HIV DNA observed at the beginning (r≤−0.67, p<0.0001) remained moderate at the end of the observation period (r = −0.53, p<0.005). In the treatment-naïve group, there were no statistically significant correlations (p≥0.23). This may be due to the small number of patients. For patients under the ART regimen, significant initial moderate inverse correlations were observed between CD4+ T cell counts and both plasma viremia (r = −0.53, p = 0.02) and total HIV DNA (r = −0.62, p = 0.01), and a stronger correlation was found with UF (r = −0.73, p<0.005). At the end of the observation period, the only correlation that is was still evident, although not statistically significant, was between CD4+ and UF (r = −0.42, p = 0.09). In the under RAL group, there was a significant moderate inverse correlation between plasma viremia and CD4+ only at the end of the observation period (r = −0.53, p = 0.03). The higher correlation between plasma viremia and the amount of HIV DNA (r≥0.61, p = 0.01) became nearly insignificant by the end of the observation period. On the contrary, the moderate inverse correlation between CD4+ and HIV DNA (r≤−0.57, p≤0.02) strengthened during the observation period becoming a strong inverse correlation (r≤−0.75, p≤0.005). In the ART & under RAL group, the only correlation that remained moderate throughout the observation period was between CD4+ and HIV DNA (r≤−0.53, p≤0.005), while the correlation between HIV-1 RNA and CD4+ or HIV DNA was reduced at the end of the study to lower values, which were not always statistically significant.

### Development and validation of 2-LTR circles in blood samples

To specifically detect 2-LTR circles in blood samples we designed a SYBR Green qPCR using primers flanking the dual-repeat cassette within the circular form, which is formed after end-to-end 5′ and 3′ LTR ligation. The primers were specific to the sequences of the M group reported in HIV Sequence Compendium 2013 [29]. Moreover, they were tested in 50 HIV-1 negative DNA samples showing no amplification in 86% of the samples (n = 43) and nonspecific amplification in the remaining 14% (n = 7). The amplification of the 2-LTR junction was linear over a 5-log range, and sensitivity allowed the detection of two copies per reaction (Ct mean value±SD: 27.37±1.11), even in the presence of 0.5 µg of bk DNA. The p2LTR standard showed a mean curve of y = −3.3287x+28.37 (n = 8), with an efficiency of 98–100%, and a linear correlation of R^2^ = 0.99. The reproducibility assayed by %CV of copy number of the standard curve was 19% in the 10∧5 to 2 copy number range. 2-LTR were measured in replicates of 0.5 µg of cellular DNA from 2 to 8 and normalized to 1 µg of DNA and to 10^4^ CD4+. To validate 2-LTR assay some randomly selected blood samples were tested (Table 4). Most of the samples (16 of 30, 53%) had very low levels of 2-LTR (<2 copies/µg DNA), while the remaining samples showed between 2 and 10 copies/µg DNA. No significant differences were found in the three groups analyzed on the basis of therapy: treatment-naïve, ART and under RAL (p = 0.379 and p = 0.770 for normalization per µg of DNA or per 10^4^ CD4+, respectively, Kruskal-Wallis test). Likewise, no significant differences were found between MDR and non-MDR groups (p = 0.741 and p = 0.207 for normalization per µg DNA or per 10^4^ CD4+ respectively, Mann-Whitney test). Due to similar PCR efficiencies for unintegrated and 2-LTR circles, we provide data regarding the 1-LTR circles and, if present, linear HIV DNA, subtracting the level of 2-LTR from all unintegrated forms.

**Table 4:** Quantification of 2-LTR circles in blood samples.

Sample	CD4+T cell count/µl	HIV-1 RNA (copies/ml)	CD4+ present in one µg of DNA	2-LTR UF HIV DNA a1-LTR + linear			2-LTR UF HIV DNA 1-LTR + linear			bMDR/non-MDR
				(copies/µg DNA)			(copies/10^4^ CD4+)			
1	739	10∧5	21199	10	13	3	5	6	1	MDR
2	426	10∧5	15685	7	11	4	4	7	3	non-MDR
3	180	10∧6	6785	<2	40	39	1	59	58	non-MDR
4	320	10∧5	9564	<2	2	1	1	2	1	MDR
5	70	10∧6	1048	<2	7	6	10	67	57	non-MDR
6	513	396	14286	<2	3	2	1	2	1	non-MDR
7	317	10∧5	14799	<2	8	7	1	5	4	non-MDR
8	596	10∧6	17131	<2	18	17	1	10	9	non-MDR
Naive, n = 8		median, IQR		<2, <2–6	10, 4–17	5, 2–15	1, 1–5	7, 3–47	4, 1–45	
9	487	<50	12184	7	8	1	6	7	1	non-MDR
10	926	<50	20541	2	3	1	1	1	0	non-MDR
11	959	<50	14528	4	4	0	3	3	0	non-MDR
12	835	<50	25060	8	9	1	3	4	1	non-MDR
13	639	10∧4	16157	3	17	14	2	10	8	non-MDR
14	321	100	13527	2	3	1	1	2	1	non-MDR
15	105	<50	5396	<2	<2	0	2	2	0	non-MDR
16	272	10∧3	9547	<2	5	4	1	5	4	non-MDR
17	192	100	5108	<2	5	4	2	9	7	non-MDR
18	227	<50	7206	8	9	1	11	13	2	non-MDR
19	462	10∧3	16709	<2	11	10	1	7	6	non-MDR
20	146	<50	3685	2	<2	0	5	3	0	non-MDR
ART, n = 12		median, IQR		2, <2–6	5, 3–9	1, 0–4	2, 1–5	5, 2–9	1, 0–6	
21	764	<50	16241	<2	<2	0	1	1	0	MDR
22	291	<50	4874	6	5	0	12	11	0	MDR
23	263	<50	4425	3	3	0	7	6	0	MDR
24	306	<50	6063	3	3	0	5	5	0	MDR
25	178	<50	2913	<2	3	2	3	10	7	MDR
26	430	<50	10519	<2	<2	0	1	1	0	MDR
27	951	<50	21195	4	7	3	2	3	1	non-MDR
28	163	10∧3	7056	<2	11	10	1	16	15	non-MDR
29	1,900	10∧3	28692	<2	<2	0	0	0	0	non-MDR
30	1,011	<50	28154	<2	3	2	0	1	1	non-MDR
Under RAL, n = 10		median, IQR		<2, <2–3	3, 1–6	0, 0–2	2, 1–6	4, 1–10	0, 0–3	
Kruskal-Wallis test				0.379	0.056	0.019	0.770	0.408	0.048	
Mann-Whitney test				0.741	0.069	0.018	0.207	0.672	0.040	

a Determined subtracting the level of 2-LTR from all unintegrated HIV DNA forms, taking into account the similar PCR efficiencies of 2-LTR and UF.

b The median (IQR) value for 2-LTR/µg DNA was 2 (<2–5) and <2 (<2–4) for MDR and non-MDR group, respectively; for 2-LTR/10^4^ CD4+ was 4 (1–7) and 2 (1–3) for the MDR and non-MDR group, respectively.

The median (IQR) value for UF HIV DNA/µg DNA was 3 (<2–5) and 7 (3–11) for the MDR and non-MDR groups, respectively; for UF HIV DNA/10^4^ CD4+ was 6 (1–9) and 5 (2–10) for the MDR and non-MDR group, respectively.

The median (IQR) value for 1-LTR + linear forms/µg DNA was 0 (0–2) and 3 (1–8) for the MDR and non-MDR group, respectively; for 1-LTR + linear forms/10^4^ CD4+ was 0 (0–1) and 2 (1–7) for the MDR and non-MDR group, respectively.

## Discussion

Total cell-associated HIV-1 DNA consists of unintegrated linear and circular 1-LTR and 2-LTR forms and integrated proviral DNA. Recent reports suggest that transcription from unintegrated HIV DNA appears to be a normal early step in HIV replication pointing to a potential role for unintegrated viral DNA in HIV-1 pathogenesis. High levels of UF can be detected *in vivo* and it is the most prevalent form of HIV DNA (2 log more than IDNA) in resting and activated CD4+ T cells [35], [36].

QPCR-based methods are now available to accurately quantify HIV DNA forms and they are widely used to explore the pathogenetic role of reservoirs, viral persistence and to monitor the effectiveness of antiviral therapy. Some of these assays have a throughput which is too low (up to 40 replicates for just a single sample) [37] and too expensive for use in large clinical trials or in routine clinical practice to complement CD4+ T cell counts and HIV-1 RNA, which are routinely used in the management of HIV-1 infected patients. Some authors have performed a concurrent measurement of total and integrated HIV DNA [16]–[18]. However, no methodology has been developed for assaying total HIV DNA and all unintegrated forms in a relatively simple manner. The aim of this work was to evaluate the reliability and usefulness of the simultaneous quantification of total and unintegrated HIV DNA forms *(TotUFsys* platform) in HIV-1 blood samples. The novelty of the strategy lies in the fact that for each sample, both measurements are obtained in a single PCR run starting from a single DNA isolated from a small amount of frozen blood, omitting the PBMC separation on ficoll-hypaque gradient. The assay uses specific primers spanning the highly conserved PBS region and its flanking sequences [28] and is unaffected by the location of the HIV-1 integration site, unlike the *Alu*-assay [17], [37]. Employing the same primers and using a single standard curve, the identical amplification efficiency and sensitivity allowed us to accurately compare the data. Moreover, it appears that the integrated level can be calculated indirectly as the difference between the total and unintegrated HIV DNA. Unlike other published reports, for the first time, the UF were separated from HMW DNA through column chromatography on silica gel, starting from a small amount of cellular DNA (5 µg) and then quantified in the LMW fraction. The phenol-chloroform DNA extraction procedure ensured a not degraded, HMW DNA, and only a slight cross-contamination of genomic DNA fragments in the LMW fraction were observed. Other faster or automated DNA extraction procedures may also be used, first thoroughly assessing genomic DNA contamination in the eluate fraction. The high rate of recovery demonstrates the feasibility of the separation method starting with DNA to measure extrachromosomal forms in HIV patients. A qPCR to specifically detect 2-LTR circles in the same DNA sample used for the quantification of total and UF HIV DNA was also developed. Due to the unique nature of the LTR-LTR junction, which can be readily assayed by PCR [8], the 2-LTR circles are often recognized as overall markers of all unintegrated forms and it has been suggested that could be a surrogate marker of HIV-1 replication, although their use remains debatable, primarily due to controversy regarding their half-life. Because of this ongoing dispute and the low 2-LTR levels (also confirmed in our group of samples), the quantification of all the unintegrated forms seemed a more correct approach to reduce the percentage of samples near the low quantification limit (from 53% to 29%). However, the method is a useful tool for the quantification of 2-LTR in DNA samples from *in vitro* experiments involving the use of purified CD4+ T cells, PBMC or macrophages, where a higher content of these forms is expected. The decision to develop both the 2-LTR and *TotUFsys* assays based on SYBR Green instead of fluorogenic probes stems from the high LTR-LTR junction sequence heterogeneity [38], and the fact that the presence of even just a single mismatched base at the 5′ end of the probe can fail to detect the target sequence and/or affect the quantifications with the risk of “false negative” results. High sensitivity (2 copies), high amplification efficiency and specificity across different clades within group M were demonstrated. In addition, no cross-reactivity with HERV, which are highly similar in terms of DNA sequence to HIV-1, was revealed in HIV-negative samples, confirming the absence of interference in very low HIV DNA copy quantification and a realistic diagnostic specificity. The accuracy of the results was improved by a standard of half-log plasmid dilutions in the low range of quantification. Reproducibility was realistic over the experimentally determined standard curve dynamic range, showing the reliability of the technical set-up over time. Moreover, to maximize assay precision in the samples with a low HIV DNA level (copy number ≤30), repetitive sampling (8 replicates) allowed us to report standard deviation, coefficient of variation and confidence interval. Reliable, simultaneous quantification of total and unintegrated HIV DNA was obtained for 195 blood samples collected from HIV-1 patients in a wide range of clinical pictures during routine laboratory monitoring. A high success rate was obtained for all the samples, even those from patients with suppressed plasma viremia, regardless of CD4+ T cell counts, or therapy. We conducted each type of analysis by considering normalization per µg of DNA as well as per 10^4^ CD4+ since they harbour most of the HIV-1 genomes detectable in blood, highlighting that inappropriate normalization may induce misleading effects and conclusion regarding the real state of patient health. Moreover, when the amount of HIV DNA is expressed for CD4+, the results could have greater relevance.

If we consider all the samples together, while there was only a marginal positive correlation between plasma viremia and the amount of HIV DNA, both total HIV DNA and unintegrated forms inversely correlated with CD4+ T cell counts. However, no significant correlation was observed between the two currently most frequently used prognostic markers: plasma viremia and CD4+ count. Within the cohort of patients, correlations were evaluated in six different clinical situations. There was consistently a significant inverse correlation between CD4+ and HIV DNA in all subsets, reaching the highest value between CD4+ and unintegrated HIV DNA and no significant correlation was found between HIV-1 RNA and CD4+, except for the treatment-naïve group. Forty-five subjects monitored for an observation period, showed the strongest correlation between CD4+ and HIV DNA and this was the only correlation that remains over time. The same conclusion could be drawn even when considering separately subjects under ART, subjects under RAL intensification or the combination of these. In particular, from moderate to very strong correlations were observed frequently between CD4+ and total HIV DNA, and almost always between CD4+ and unintegrated HIV DNA. These analyses highlight the limited correlation between CD4+ and plasma viremia in patients under classical ART or/and ART plus an integrase inhibitor agent such as Raltegravir and show that the correlation is often lost after effective ART. In general, we found that in our cohort of patients representing different clinical situations, there was a weak or no correlation between CD4+ and viremia. However, we found a high inverse correlation between CD4+ and HIV DNA with the strongest correlations for unintegrated forms.

## Conclusions

The use of a distinctive and well-performing workflow and a layout of PCR plates (*TotUFsys* platform), allowed us to obtain in less than two working days, HIV DNA copy number per µg of DNA or 10^4^ CD4+ for 12 HIV-1 patients. We developed a practical method able to simultaneously measure total and unintegrated HIV DNA as well as indirectly integrated provirus, in a wide range of clinical situations typical of HIV-1 infection, such as treatment-naive, under effective/suboptimal ART, new drug regimes, MDR and or co-infected patients. Because the assay makes use of frozen whole blood specimens, it has broad applications and is well-suited for a large series of sequential samples collected within clinical trials/vaccination protocols. A careful choice of the most suitable DNA extraction method makes it possible to easily adapt our assay to alternative sample types such as tissue biopsies, purified CD4+ T cells, PBMC or macrophages from *in vitro* experiments, and on the same specimen collected for routine plasma viremia determination, after removal of the plasma for the HIV-RNA assay. Our findings support the quantification of total and unintegrated HIV DNA as an additional or alternative tool to traditional assays to estimate the state of viral infection, the risk of disease progression and to monitor the effects of therapy (suppressive or new treatments), providing useful data that could influence decisions whether to initiate, change, intensify or simplify the ART. Moreover, the newly developed *TotUFsys* platform is relatively fast and less labor intensive than other already existing quantification assays.

## Materials and Methods

The essential steps of the procedure for the quantification of total and unintegrated HIV DNA forms in 12 samples, from frozen blood until data analysis is illustrated in Figure 1.

The methods were carefully developed following the validation parameters (specificity, limit of quantification (QL) and detection (DL), linear range of quantification, reproducibility and repeatability) specified in the Guidelines for the Validation of Analytical Procedures (http://www.ich.org/fileadmin/Public_Web_Site/ICH_Products/Guidelines/Quality/Q2_R1/Step4/Q2_R1__Guideline.pdf).

### Patients and blood samples

Fifty-nine adult HIV-1 positive patients, who reported to the reference hospital (Azienda Ospedaliera Ospedali Riuniti Marche Nord - Presidio Ospedaliero San Salvatore, Pesaro, Italy) from January 2009 until May 2011 for routine blood tests, provided from a single sample to nine blood samples for a total of 195 specimens. All subjects were asked to sign a written informed consent for the collection and storage of their blood samples for research purposes, according to Declaration of Helsinki principles. The study was approved by the San Salvatore Hospital ethics committee.

### Quantification of plasma viremia and CD4+ T cell counts

Plasma obtained from blood samples in EDTA was frozen at −80°C until tested.

The viral load in plasma was quantified using the Artus HI Virus-1 QS-RGQ Kit. The kit is a ready-to-use system for the detection of HIV-1 RNA using PCR on Rotor-Gene Q Instruments. Sample preparation and assay setup make use of the QIAsymphony SP/AS instruments (Qiagen), according to the manufacturer's instructions.

Lymphocyte surface phenotypes and CD4+ lymphocyte counts were determined using flow cytometry analysis by Immunotech-Beckman Coulter (Marseille, France).

### Nucleic acid extraction

For each sample, the cellular DNA was isolated from leukocytes (WBC) from 3 or 4 ml of peripheral blood according to the previously described method [28]. Briefly, after incubation of the WBC pellet for 45 min at 37°C in a lysis buffer, the DNA was purified by phenol extraction followed by ethanol precipitation and RNase treatment. Isolated DNAs were quantified by NanoDrop ND-1000 Spectrophotometer (NanoDrop Technologies, Wilmington, DE, USA) and the absorbance ratios of 260/280 and 260/230 were used to control the purity of the samples: all samples had a ratio of about 1.8 and 2.0 respectively, and are accepted as “pure” DNA. A mean DNA recovery of 20±7 µg/ml of blood was obtained for a total of 60 or 80 µg of DNA/blood sample, more than adequate for the quantification of all HIV DNA forms. One aliquot of HIV-1 negative blood was extracted in each experiment, together with the clinical samples to monitor extraction procedure. Ten µg of DNA were mixed with 1.5 volume of hydrogen peroxide solution and incubated at 37°C for 30 min prior to ethanol precipitation and re-suspension to obtain a theoretical concentration of 100 ng/µl. The DNA were then quantified again. This step was performed to improve low copy detection of the total HIV DNA and 2-LTR circles on a consistent background of high molecular weight DNA in PCR experiments (up to 1 µg/PCR).

### Isolation of unintegrated HIV DNA

The extrachromosomal HIV DNA was purified from 5 µg of cellular DNA using the QIAprep miniprep kit (Qiagen) according to the manufacturer's instructions and the recommended modifications were used for the isolation of low-copy number plasmids. Moreover, we made some further changes in the amount of the supernatant loaded in each column (600 µl of 850 µl total, corresponding to 71% of the processed DNA, 3529 ng) and the volume of elution (106 µl of buffer EB). Two separate purifications were performed for each sample and the eluate fractions containing extrachromosomal forms (low molecular weight, LMW fraction DNA), were combined at the end of the procedure. To monitor for cross-contamination, one sample of H_2_O in place of DNA and one HIV-1 negative DNA were processed every twelve samples.

### Oligonucleotide primers

The primers were selected and analyzed using the Oligo Primer Analysis software (version 6.65; Cascade, CO, USA). The forward primer PBSf (5′-TAGCAGTGGCGCCCGA-3′) and the reverse primer PBSr (5′-TCTCTCTCCTTCTAGCCTCCGC-3′); the forward primer 2LTRf (5′-TAGTGTGTGCCCGTCTGT-3′) and the reverse primer 2LTRr (5′-TGTGTAGTTCTGCCAATCAG-3′); the forward primer EXgf (5′-CCGCTGTATCACAAGGGCTGGTACC-3′) and the reverse primer EXgr (5′-GGAGCCCGTGTAGAGCATGACGATC-3′); the forward primer ACTf (5′-CCGCCCGTCCACACCC-3′) and the reverse primer ACTr (5′-CTGACCCATGCCCACCATCA-3′) were purchased from Sigma-Genosys and maintained at −20°C at a concentration of 100 µM in TE 10-1 mM, pH 8.0, in single-use aliquots.

### Construction of plasmid and standard curves

The use of the pPBS plasmid (161 bp-PBS fragment derived from HIV-1 PNL4-3 vector cloned into the pGEM-T vector) as a reference standard was previously validated [28].

The 2-LTR plasmid (p2LTR) was obtained by cloning a 176 bp of LTR-LTR junction within the circular form in a pGEM-T vector.

The 13 Kb exogenous plasmid (pEXg) was obtained by cloning a 225 bp fragment of a plant gene (GenBank accession no. U16123) in an appropriate plasmid (PINCO: 12889 bp) [39].

The cloned fragment sequences were confirmed using the automatic sequencer ABI Prism 310 Genetic Analyzer (Applied Biosystems, Foster City, CA, USA).

To determine the exact copy number, the linearized plasmids were accurately quantified with the NanoDrop ND-1000 Spectrophotometer. Standard curves (pPBSstd, p2LTRstd and pEXgstd) were constructed with 10-fold and half-log plasmid serial dilutions, in a range from 10∧5 to 10, including 2 molecules. Dilutions in TE buffer were freshly prepared for each experiment from aliquots of 10∧9 copies stored at −80°C. The standard curve (ACTstd) used for the quantification of a 177 bp fragment of the β-actin housekeeping gene (ACT, GenBank accession no. NM_001101.3), was made freshly for each experiment with 10- and 2-fold serial dilutions of a reference genomic DNA ranging from 1000 to 0.01 ng.

### SYBR Green I real time PCR

The organization of the *TotUFsys* platform for the quantification of HIV DNA forms (total and unintegrated) is described in the paragraph below. QPCR of various targets was set up in a final volume of 100 µl using 96-well plates. 0.5–1.0 µg of cellular DNA or the equivalent quantity in µl of LMW fraction DNA was added to the mixture containing 50 µl of 2× master mix Hot-Rescue Real Time PCR Kit-SG (Diatheva srl, Fano, Italy) and 100 nM of each primer. For the pEXg and β-actin, a variable quantity of DNA was assayed on the basis of the specific PCR experiment. The amplification profile for total HIV DNA, unintegrated HIV DNA, pEXg and β-actin was as follows: one cycle of 10 min at 95°C to activate the Hot-Rescue DNA polymerase followed by 40 cycles in two steps, consisting of 15 sec at 95°C and 35 sec at 68°C, while for 2-LTR circles one cycle of 15 min at 95°C followed by 40 cycles of three steps consisting of 95°C for 15 sec, annealing at 60°C for 20 sec and extension at 72°C for 35 sec. The fluorescence intensity of the products was measured at the end of each cycle and post-PCR melt curve analysis was performed to detect primer-dimers or other non-specific products and to confirm the specificity of the target. Amplification, data acquisition and analysis were carried out using an Applied Biosystems 7500 Real-Time PCR instrument with the Sequence Detection System software package (version 1.4.0). Three (for copy numbers ≥10) or six replicates (for copy numbers below 10) of standard scalar dilutions were included in each plate. Standard curves were created automatically and accepted when the slopes were between −3.40 and −3.26 (97–100% efficiency) and the minimum value of the correlation coefficient (R^2^) was 0.98. The percentage of amplification efficiency was calculated as (10^∧^(−1/slope)−1)×100. In all experiments, negative controls containing water (NTC) or HIV-1 negative DNA were tested.

### Data analysis of quantification of total, unintegrated (*TotUFsys* platform) and 2-LTR HIV DNA forms

The *TotUFsys* platform was performed essentially exploiting the whole blood leukocyte pbs-rtPCR assay [28] with the following improvements:

- a more precise standard of half-log serial dilutions in the low range of quantification (from 10∧3 to 10, and 2-copy dilution) rather than the broad dynamic range that is normally used, unless otherwise specified.
- Each clinical sample was analyzed in triplicate. The first PCR (1^st^ qPCR) consisted of two wells containing 0.5 µg each plus one well containing 1.0 µg of DNA or the equivalent quantity in µl of the LMW fraction DNA. The amount of 0.5 µg was increased by doubling to 1.0 µg to ensure the detection of the target even in the low copy number (near the QL). The copy number measured for each replicate was obtained by interpolation of the Ct value from the standard and if this was quantified over 30 copies/PCR determination (coefficient of variation 21%), the result for each sample was given adding up the copy number from the two 0.5 µg replicates and expressed as HIV DNA copy number/µg of DNA or LMW fraction DNA.
- A second PCR (2^nd^ qPCR) performed for an HIV DNA datum quantified below 30 copies/PCR determination, and consisting of:
  c.1) six 0.5 µg (for a total of 3 µg) replicates for samples which in the 1^st^ qPCR had been quantified in the range between 30 to 2 copies/PCR determination. The result was given dividing by 4 the sum of the copy number from a total of eight replicates (two 0.5 µg replicates in the 1^st^ qPCR+six 0.5 µg replicates in the 2^nd^ qPCR) and expressed as HIV DNA copy number/µg of DNA or LMW fraction DNA;
  c.2) three 0.5 µg (for a total of 1.5 µg) replicates and three 1.0 µg (for a total of 3.0 µg) replicates for samples which in the 1^st^ qPCR had been quantified near or detected below the QL (<2 copies/PCR replicate). After excluding the presence of inhibitors adding 2 or 10 pPBS standard copies in a spike-PCR, the result was given by adding copy numbers from the quantifiable replicates for a total of 6.5 µg (∼10∧6 WBC, assuming 7.0 pg of DNA content per human diploid genome as conversion factor [40]) of DNA or LMW fraction DNA processed (two 0.5 µg replicates plus one 1.0 µg replicate in the 1^st^ qPCR+three 0.5 µg replicates plus three 1.0 µg replicates in the 2^nd^ qPCR) and expressed as HIV DNA copy number/6.5 µg of DNA or LMW fraction DNA. For subsequent analysis these data were then expressed to one µg of DNA or LMW DNA fraction.
- HIV DNA copy number was also expressed as copies/10^4^ CD4+ T cells, assuming that 142857 cells are present in one µg of DNA. The following formula was used for each sample:
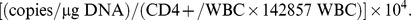
Taking into account WBC number/ml of whole blood, the result can be reported as the HIV DNA copy number/ml of whole blood, with the formula: HIV DNA copy no. per µg DNA×WBC no. per ml/142857 cells [28].
- The number of PCR cycles was reduced from 45 to 40, and the data collection of the first ten cycles is omitted (the Ct value is in the range 1 to 30) for a more accurate threshold set up.

### Quantification of integrated HIV DNA measurement

Integrated HIV DNA was obtained indirectly, subtracting the unintegrated HIV DNA determined directly in the LMW fraction from total HIV DNA which was also determined directly in total cellular DNA with the formula: total HIV DNA copy number/µg DNA – UF copy number/µg of LMW fraction DNA. Integrated HIV DNA was then normalized to 10^4^ CD4+ T cells. We also included examples of indirect calculation of integrated proviral DNA showing the feasibility of this approach.

### Statistical analysis

The Ct values of the standard dilutions were exported to a Microsoft Excel worksheet for the calculation of averages, standard deviations (SD) and coefficients of variation (CV%) in order to evaluate the method's accuracy.

The data are presented as medians, range (minimum to maximum) and interquartile range (IQR, 25th to 75th percentile). All comparisons between two different groups were performed using the nonparametric Mann-Whitney U test for unpaired data or the nonparametric Kruskal-Wallis test for comparison between more than two groups. The correlation between two parameters was determined using Spearman's correlation coefficient. For samples below the limit of quantification (QL), an imputed value corresponding to ½ QL was used for statistical analysis [41]. Statistical significance was accepted for p values below 0.05.

## Supporting Information

Figure S1 **Primer specificity for HIV-1 group M** http://www.hiv.lanl.gov/content/sequence/HIV/COMPENDIUM/2013compendium.html **.**
(PDF)

Figure S2 **Gel electrophoresis analysis of HMW DNA and Marker II eluate fractions using QIAprep miniprep kit (Qiagen).**
(PDF)

Table S1 **Diagnostic specificity.**
(PDF)

Table S2 **pPBS standard curve in assence or presence of HIV-1 negative human DNA.**
(PDF)

Table S3 **Ct value and copy number.**
(PDF)

Table S4 **Method reproducibility calculated by the percentage of the coefficient of variation for Ct value (A) and for copy number (B).**
(PDF)

Table S5 **Quantification of total HIV DNA copy number in three different samples tested in three separate experiments.**
(PDF)

Table S6 **Cross-contamination level of HMW DNA measured in eluate fraction by qPCR of β-actin housekeeping gene.**
(PDF)

Table S7 **Recovery test of extrachromosomal forms.**
(PDF)

Table S8 **Characteristics of total, unintegrated and integrated HIV DNA measurements in samples.**
(PDF)

Table S9 **Correlations between study parameters in blood samples.**
(PDF)

Table 10 **Correlation between study parameters in patients at the beginning and the end of the observation period.**
(PDF)
