# Supplementary material for: A Real Time PCR Platform for the Simultaneous Quantification of Total and Extrachromosomal HIV DNA Forms in Blood of HIV-1 Infected Patients
Source: PLoS One. 2014 Nov 3;9(11):e111919. doi: 10.1371/journal.pone.0111919 (PMC4218859; doi:10.1371/journal.pone.0111919)

Figure S1

Primer specificity for HIV-1 group M

http://www.hiv.lanl.gov/content/sequence/HIV/COMPENDIUM/2013compendium.html

HIV Sequence Compendium 2013

Editors

Brian Foley
Los Alamos National Laboratory

Thomas Leitner
Los Alamos National Laboratory

Cristian Apetrei
University of Pittsburgh

Beatrice Hahn
University of Pennsylvania

Ilene Mizrachi
National Center for Biotechnology Information

James Mullins
University of Washington

Andrew Rambaut
University of Edinburgh

Steven Wolinsky
Northwestern University

Bette Korber
Los Alamos National Laboratory

Project Officer

Stuart Shapiro
Division of AIDS
National Institute of Allergy and Infectious Diseases

Los Alamos HIV Sequence Database and Analysis Staff

Werner Abfalterer, Mira Dimitrijevic, Shihai Feng, Will Fischer, Bob Funkhouser, Peter Hraber, Jennifer Macke, James J. Szinger, Hyejin Yoon

This publication is funded by the Division of AIDS, National Institute of Allergy and Infectious Diseases, through interagency agreement IAA Y1-AI-8309-1 "HIV/SIV Database and Analysis Unit" with the U.S. Department of Energy.

Published by
Theoretical Biology and Biophysics
Group T-6, Mail Stop K710
Los Alamos National Laboratory
Los Alamos, New Mexico 87545 U.S.A.

LA-UR-13-26007

http://www.hiv.lanl.gov/

II

HIV-1/SIVcpz Complete Genomes

Contents

II-1 Introduction 9
II-2 Annotated features 10
II-3 Sequences 13
II-4 Alignments 18

II-1 Introduction

While the "web-alignment" this year has grown to 2229 full-length genomes, the compendium alignment herein has strict page limitations. The selection here was made such that subtypes A-G, CRF's 01 and 02, and CPZ are represented with one sequence from most countries where the subtype has been sequenced. Priority was given to more recently sampled sequences to reflect the current pandemic diversity. For the rare subtypes H–K and groups N–P most available sequences are included, and for CRF's 03 and up, one representative is included. Unique recombinant forms and unclassified sequences are not included. This resulted in 199 sequences of HIV-1/SIVcpz from all over the world.

The HXB2 sequence (accession K03455) is the master sequence in this alignment. This is also the genome coordinate standard used throughout the HIV Database. The alignment was generated by MAFFT v7.043 (E-INS-i with gap open penalty 2.0). The alignment was subsequently codon-aligned using GeneCutter, followed by a few manual edits to fix obvious misalignments. The alignment presented cannot be considered an "optimal alignment" to any single criterion; it is a compromise between optimal alignment, readability, and codon alignment. Most gaps have been introduced in multiples of 3 bases to maintain open reading frames when the alignment is translated.

Also part of this nucleotide alignment is a translation to protein sequence based on the HXB2 sequence; the HIV genome has many overlapping coding regions, and all are shown. For more complete annotation of functional domains, see the protein sequence alignments in Chapter V.

Forward primer PBS 5'-TAGCAGTGGCGCCCGA-3'

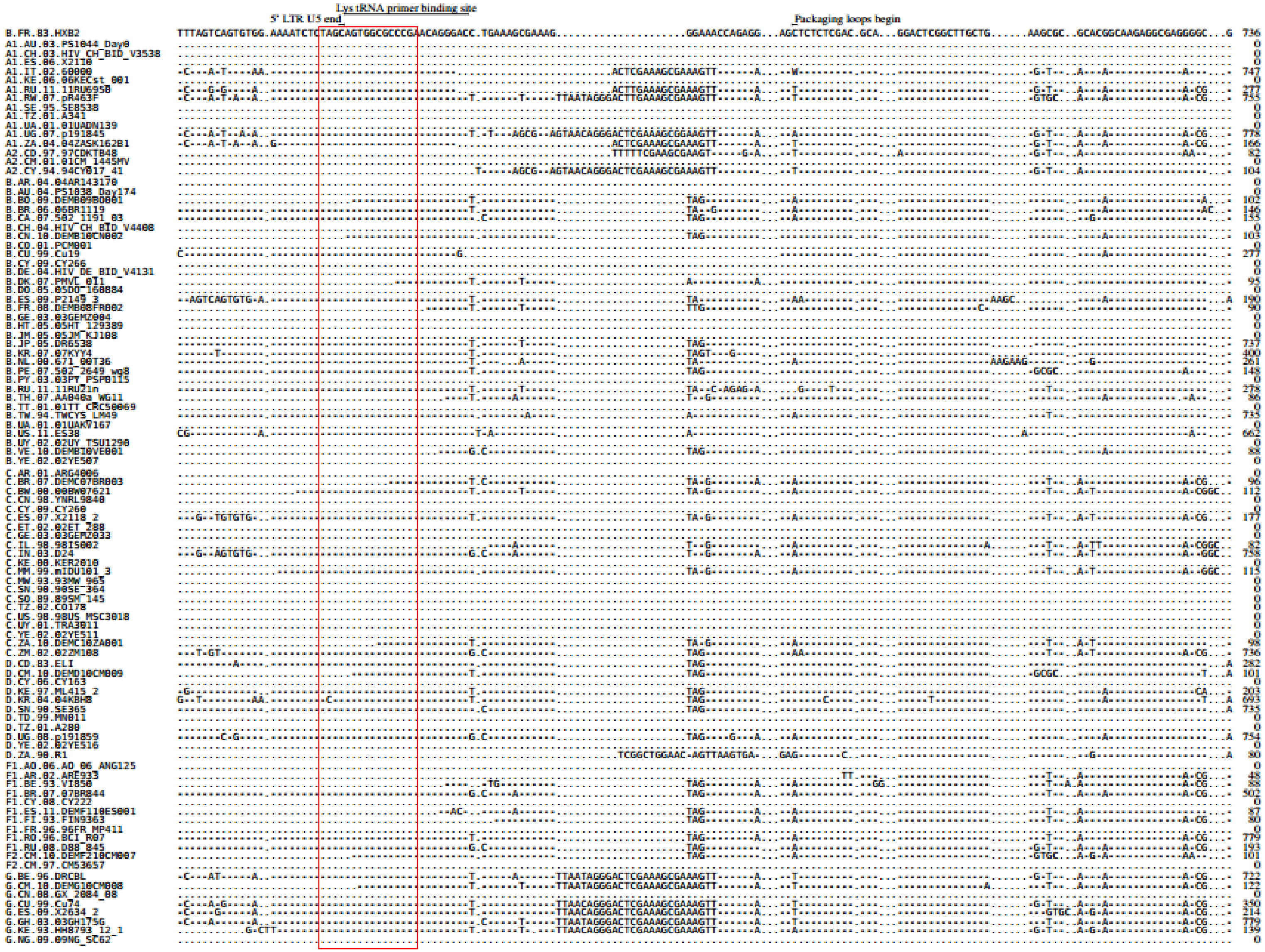

Forward primer PBS 5'-TAGCAGTGGCGCCCGA-3'

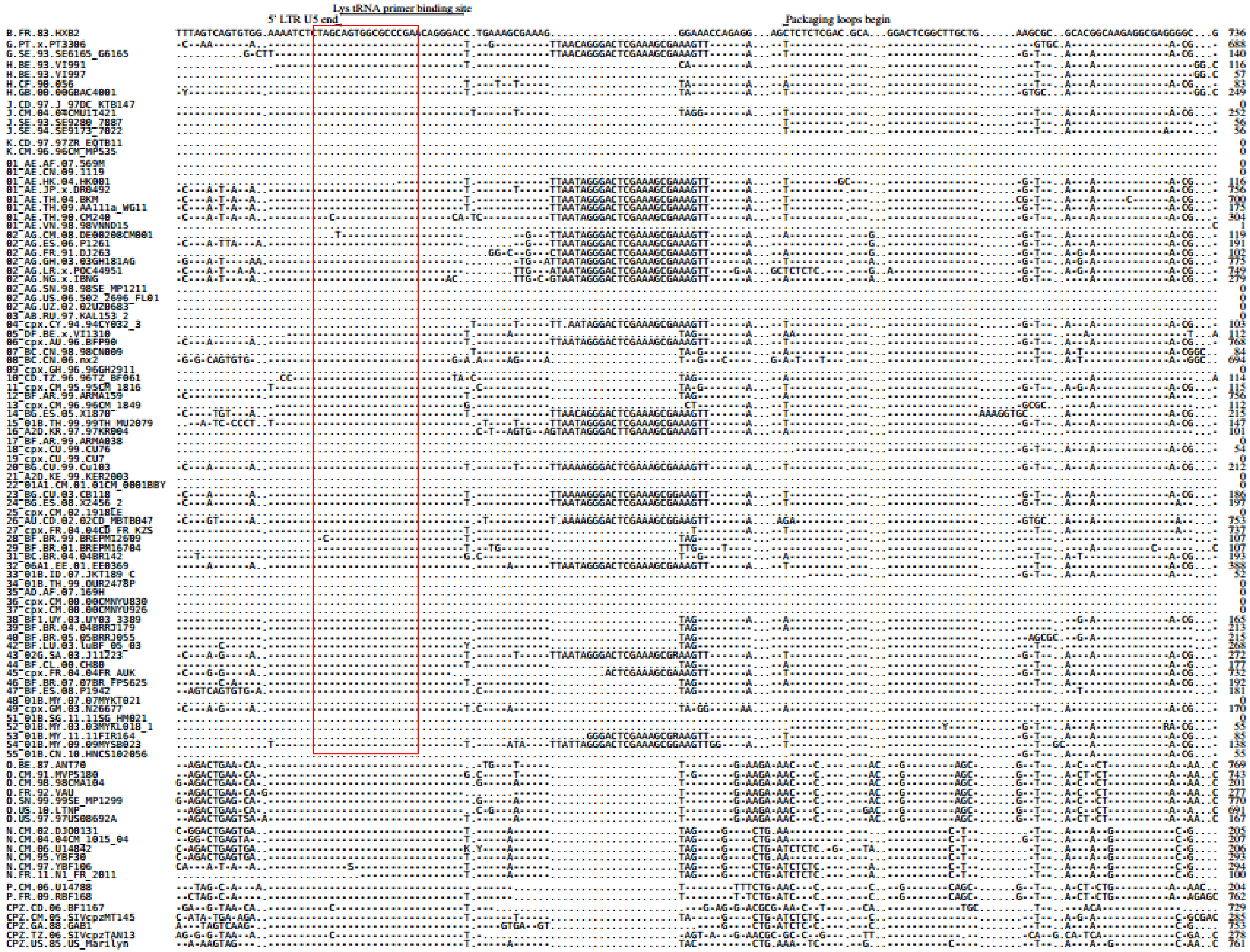

Reverse primer PBS 5'-TCTCTCTCCTTCTAGCCTCCGC-3'

Packaging loops end — Gag and Gag-Pol start

Sequence	Alignment	Position
B.FR.83.HXB2	GC...GACTGGTGAGTACGCCAAAAA........TTTTGACTAGCGGAGG......CTAGAAGG...................AGAGAG..ATGGGTGCGAGAGCGTCAGTATTAAGCGGGGGAGAATTAGATCGATGGGAAAAAATTCGGTTAAGGCCAGGGGGAAAG	867
Gag	M G A R A S V L S G G E L D R W E K I R L R P G G K	
A1.AU.03.PS1044_Day0	[illegible]	78
A1.CH.03.HIV_CH_BID_V3538	[illegible]	78
A1.ES.06.X2110	[illegible]	111
A1.IT.02.60000	[illegible]	878
A1.KE.06.06KECst_001	[illegible]	79
A1.RU.11.11RU6950	[illegible]	409
A1.RW.07.pR463F	[illegible]	888
A1.SE.95.SE8538	[illegible]	72
A1.TZ.01.A341	[illegible]	72
A1.UA.01.01UADN139	[illegible]	72
A1.UG.07.p191845	[illegible]	906
A1.ZA.04.04ZASK162B1	[illegible]	299
A2.CD.97.97CDKTB48	[illegible]	214
A2.CM.01.01CM_1445MV	[illegible]	72
A2.CY.94.94CY017_41	[illegible]	236
B.AR.04.04AR143170	[illegible]	74
B.AU.04.PS1038_Day174	[illegible]	78
B.BO.09.DEMB09BO001	[illegible]	236
B.BR.06.06BR1119	[illegible]	284
B.CA.07.502_1191_03	[illegible]	288
B.CH.04.HIV_CH_BID_V4408	[illegible]	78
B.CN.10.DEMB10CN002	[illegible]	233
B.CO.01.PCM001	[illegible]	72
B.CU.99.Cu19	[illegible]	408
B.CY.09.CY266	[illegible]	78
B.DE.04.HIV_DE_BID_V4131	[illegible]	78
B.DK.07.PMVL_011	[illegible]	226
B.DO.05.05DO_160884	[illegible]	72
B.ES.09.P2149_3	[illegible]	323
B.FR.08.DEMB08FR002	[illegible]	223
B.GE.03.03GEMZ004	[illegible]	72
B.HT.05.05HT_129389	[illegible]	72
B.JM.05.05JM_KJ108	[illegible]	72
B.JP.05.DR6538	[illegible]	871
B.KR.07.07KYY4	[illegible]	333
B.NL.00.671_00T36	[illegible]	393
B.PE.07.502_2649_wg8	[illegible]	279
B.PY.03.03PY_PSP0115	[illegible]	72
B.RU.11.11RU21n	[illegible]	411
B.TH.07.AA040a_WG11	[illegible]	219
B.TT.01.01TT_CRC50069	[illegible]	72
B.TW.94.TWCYS_LM49	[illegible]	866
B.UA.01.01UAKV167	[illegible]	72
B.US.11.ES38	[illegible]	790
B.UY.02.02UY_TSU1290	[illegible]	72
B.VE.10.DEMB10VE001	[illegible]	219
B.YE.02.02YE507	[illegible]	72
C.AR.01.ARG4006	[illegible]	72
C.BR.07.DEMC07BR003	[illegible]	229
C.BW.00.00BW07621	[illegible]	244
C.CN.98.YNRL9840	[illegible]	78
C.CY.09.CY260	[illegible]	78
C.ES.07.X2118_2	[illegible]	310
C.ET.02.02ET_288	[illegible]	72
C.GE.03.03GEMZ033	[illegible]	72
C.IL.98.98IS002	[illegible]	213
C.IN.03.D24	[illegible]	888
C.KE.00.KER2010	[illegible]	72
C.MM.99.mIDU101_3	[illegible]	246
C.MW.93.93MW_965	[illegible]	72
C.SN.90.90SE_364	[illegible]	72
C.SO.89.89SM_145	[illegible]	72
C.TZ.02.CO178	[illegible]	72
C.US.98.98US_MSC3018	[illegible]	72
C.UY.01.TRA3011	[illegible]	72
C.YE.02.02YE511	[illegible]	72
C.ZA.10.DEMC10ZA001	[illegible]	231
C.ZM.02.02ZM108	[illegible]	866
D.CD.83.ELI	[illegible]	413
D.CM.10.DEMD10CM009	[illegible]	233
D.CY.06.CY163	[illegible]	78
D.KE.97.ML415_2	[illegible]	333
D.KR.04.04KBH8	[illegible]	826
D.SN.90.SE365	[illegible]	866
D.TD.99.MN011	[illegible]	85
D.TZ.01.A280	[illegible]	72
D.UG.08.p191859	[illegible]	885
D.YE.02.02YE516	[illegible]	72
D.ZA.90.R1	[illegible]	213
F1.AO.06.AO_06_ANG125	[illegible]	0
F1.AR.02.ARE933	[illegible]	176
F1.BE.93.VI850	[illegible]	222
F1.BR.07.07BR844	[illegible]	630
F1.CY.08.CY222	[illegible]	78
F1.ES.11.DEMF110ES001	[illegible]	218
F1.FI.93.FIN9363	[illegible]	208
F1.FR.96.96FR_MP411	[illegible]	78
F1.RO.96.BCI_R07	[illegible]	906
F1.RU.08.D88_845	[illegible]	323
F2.CM.10.DEMF210CM007	[illegible]	232
F2.CM.97.CM53657	[illegible]	72
G.BE.96.DRCBL	[illegible]	852
G.CM.10.DEMG10CM008	[illegible]	251
G.CN.08.GX_2084_08	[illegible]	104
G.CU.99.Cu74	[illegible]	480
G.ES.09.X2634_2	[illegible]	344
G.GH.03.03GH175G	[illegible]	910
G.KE.93.HH8793_12_1	[illegible]	269
G.NG.09.09NG_SC62	[illegible]	74

Reverse primer PBS 5'-TCTCTCTCCTTCTAGCCTCCGC-3'

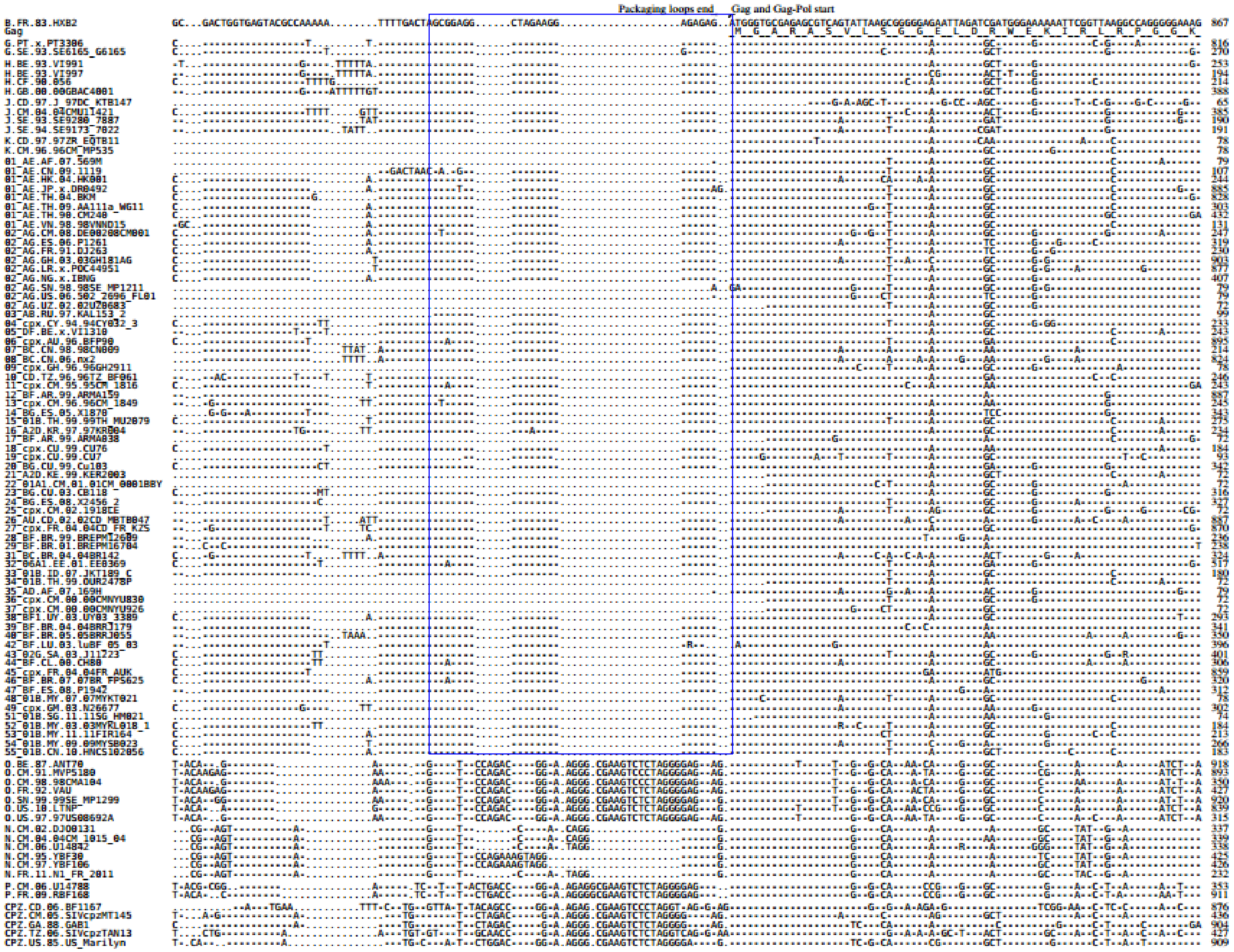

Supplement: Figure S1 — Primer specificity for HIV-1 group M http://www.hiv.lanl.gov/content/sequence/HIV/COMPENDIUM/2013compendium.html . (PDF) [file pone.0111919.s001.pdf]
