# Supplementary material for: A Real Time PCR Platform for the Simultaneous Quantification of Total and Extrachromosomal HIV DNA Forms in Blood of HIV-1 Infected Patients
Source: PLoS One. 2014 Nov 3;9(11):e111919. doi: 10.1371/journal.pone.0111919 (PMC4218859; doi:10.1371/journal.pone.0111919)
Supplement: Figure S2 — Gel electrophoresis analysis of HMW DNA and Marker II eluate fractions using QIAprep miniprep kit (Qiagen). (PDF) [file pone.0111919.s002.pdf]

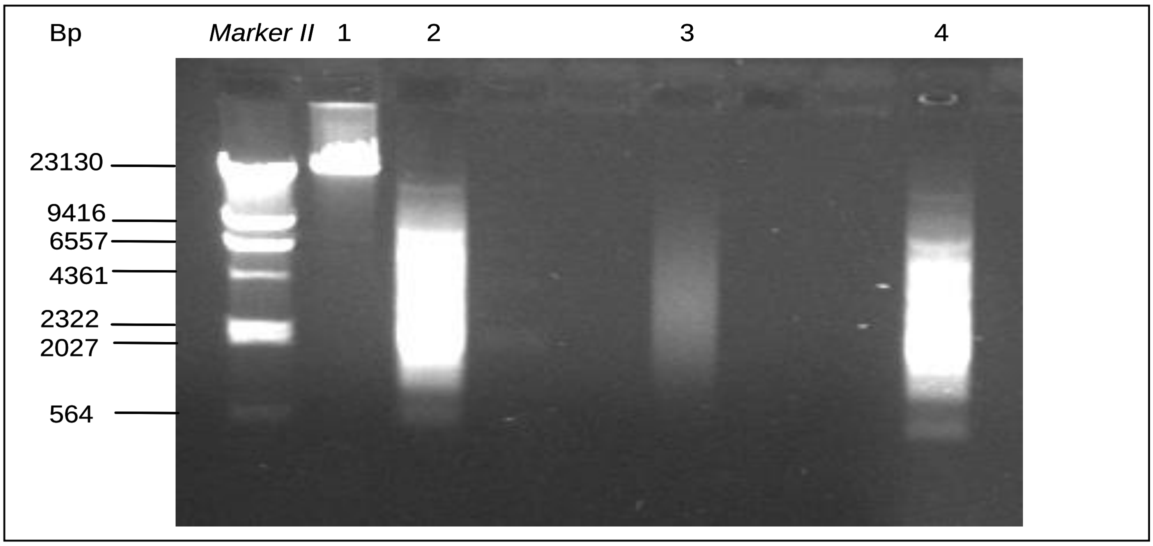

Figure S2 Gel electrophoresis analysis of HMW DNA and Marker II eluate fractions using QIAprep miniprep kit (Qiagen). Only one representative experiment is shown.
Lane1: 5 µg of cellular genomic DNA extracted with phenol procedure .
Lane 3: eluate fraction of HMW DNA sample alone (5 µg).
Lane 4: eluate fraction of Marker II alone (5 µg). The absence of DNA fragment > 10 kb was evident.
In the lane 2 we showed eluate fraction of a DNA pool of 2.5 µg of DNA plus an equal amount of Marker II to better simulate an HIV-1 infected sample. Only DNA <10 kb (about genome size of HIV-1) was recovered.
All eluate fractions show the absence of DNA fragments > 10 Kb.
The images were acquired using a Gel Doc TM 1000 Video Gel Documentation System with Molecular Analysts software version 2.1 (Bio-Rad, Hercules, CA, USA), exceeding the saturation to better reveal the rate of DNA degradation.
