# Supplementary material for: A Real Time PCR Platform for the Simultaneous Quantification of Total and Extrachromosomal HIV DNA Forms in Blood of HIV-1 Infected Patients
Source: PLoS One. 2014 Nov 3;9(11):e111919. doi: 10.1371/journal.pone.0111919 (PMC4218859; doi:10.1371/journal.pone.0111919)
Supplement: Table S1 — Diagnostic specificity. (PDF) [file pone.0111919.s003.pdf]

Table S1 Diagnostic specificity

Sample	Dissociation curve	Ct	Copy no./PCR
n1	Primer-dimer (A)	undet	
n2	Primer-dimer	25.56	
n3	HIV-1 specific peak (B)	26.21	1
n4	Primer -dimer plus HIV-1 specific peak (C)	26.8	
n5	HIV-1 specific peak	26.2	1
n6	Primer-dimer	24.88	
n7	No amplification (D)	undet	
n8	Primer-dimer	27.26	
n9	HIV-1 specific peak	25.99	<1
n10	Primer -dimer plus HIV-1 specific peak	24.92	
n11	HIV-1 specific peak	25.14	1
n12	Primer-dimer	25.63	
n13	Primer-dimer	23.35	
n14	Primer -dimer plus HIV-1 specific peak	24.53	
n15	Primer-dimer	24.75	
n16	Primer -dimer plus HIV-1 specific peak	24.63	
n17	Primer -dimer plus HIV-1 specific peak	23.36	
n18	Primer-dimer	24.75	
n19	Primer -dimer plus HIV-1 specific peak	23.78	
n20	Primer-dimer	23.78	
n21	HIV-1 specific peak	25.78	<1
n22	Primer-dimer	26.62	
n23	Primer -dimer plus HIV-1 specific peak	22.34	
n24	Primer-dimer	25.33	
n25	Primer-dimer	23.44	
n26	Primer-dimer	24.12	
n27	Primer-dimer	22.46	
n28	Primer-dimer	26.33	
n29	No amplification	undet	
n30	Primer-dimer	25.3	
n31	Primer-dimer	27.69	
n32	No amplification	undet	
n33	HIV-1 specific peak	26.62	<1
n34	HIV-1 specific peak	25.66	<1
n35	No amplification	undet	
n36	Primer -dimer plus HIV-1 specific peak	26.8	
n37	No amplification	undet	
n38	Primer-dimer	26.3	
n39	No amplification	undet	
n40	Primer -dimer plus HIV-1 specific peak	24.54	
n41	HIV-1 specific peak	26.95	<1
n42	Primer-dimer	26.9	
n43	HIV-1 specific peak	25.43	<1
n44	HIV-1 specific peak	26.59	<1
n45	Primer -dimer plus HIV-1 specific peak	26.00	
n46	No amplification	undet	
n47	HIV-1 specific peak	27.28	<1
n48	HIV-1 specific peak	27.76	<1
n49	HIV-1 specific peak	26.22	1
n50	HIV-1 specific peak	27.44	<1
n51	No amplification	undet	
n52	Primer-dimer	undet	
n53	No amplification	undet	
n54	No amplification	undet	
n55	No amplification	undet	
n56	Primer-dimer	27.6	
n57	Primer-dimer	24.56	
n58	No amplification	undet	
n59	Primer-dimer	29.8	
n60	Primer -dimer plus HIV-1 specific peak	29.7	
n61	No amplification	undet	
n62	No amplification	undet	
n63	No amplification	undet	
n64	Primer -dimer plus HIV-1 specific peak	24.74	
n65	Primer-dimer	27.68	
n66	Primer-dimer	28.15	
n67	Primer-dimer	26.23	
n68	Primer-dimer	26.85	
n69	Primer-dimer	26.76	
n70	No amplification	undet	
n71	No amplification	undet	
n72	No amplification	undet	
n73	No amplification	undet	
n74	No amplification	undet	
n75	No amplification	undet	
n76	No amplification	undet	
n77	No amplification	undet	
n78	Primer-dimer	29.45	
n79	No amplification	undet	
n80	No amplification	undet	
n81	HIV-1 specific peak	27.85	<1
n82	HIV-1 specific peak	26.29	1
n83	HIV-1 specific peak	28.23	<1
n84	Primer-dimer	29.08	
n85	HIV-1 specific peak	26.97	<1
n86	No amplification	undet	
n87	HIV-1 specific peak	27.6	<1
n88	HIV-1 specific peak	26.9	<1
n89	HIV-1 specific peak	26.92	<1
n90	No amplification	undet	
n91	HIV-1 specific peak	29.09	<1
n92	No amplification	undet	
n93	HIV-1 specific peak	undet	undet
n94	Primer -dimer plus HIV-1 specific peak	26.49	
n95	Primer-dimer	26.76	
n96	HIV-1 specific peak	26.3	<1
n97	No amplification	undet	
n98	Primer-dimer	25.27	
n99	Primer -dimer plus HIV-1 specific peak	26.76	
n100	Primer -dimer plus HIV-1 specific peak	25.12	
n101	Primer-dimer	22.87	
n102	Primer-dimer	24.67	
n103	HIV-1 specific peak	26.9	<1
n104	Primer-dimer	29.93	
n105	Primer -dimer plus HIV-1 specific peak	20.82	
n106	Primer -dimer plus HIV-1 specific peak	25.32	
n107	No amplification	undet	
n108	Primer -dimer plus HIV-1 specific peak	25.1	
n109	Primer-dimer	26.11	
n110	Primer-dimer	25.67	
n111	Primer-dimer	25.57	
n112	Primer-dimer	25.63	
n113	Primer-dimer	22	
n114	Primer-dimer	27.03	
n115	No amplification	undet	
n116	Primer-dimer	27.02	
n117	HIV-1 specific peak	26.1	<1
n118	Primer-dimer	26.11	
n119	HIV-1 specific peak	27.2	<1
n120	No amplification	undet	
n121	Primer-dimer	28.29	
n122	Primer-dimer	25.35	
n123	HIV-1 specific peak	25.82	1
n124	No amplification	undet	
n125	Primer-dimer	26.55	
n126	Primer-dimer	26.69	
n127	Primer-dimer	27.17	
n128	Primer-dimer	26.6	
n129	Primer-dimer	26.53	
n130	No amplification	undet	
n131	No amplification	undet	
n132	Primer -dimer plus HIV-1 specific peak	27.00	
n133	HIV-1 specific peak	25.6	1
n134	HIV-1 specific peak	25.93	1
n135	Primer -dimer plus HIV-1 specific peak	26.85	
n136	Primer-dimer	25.97	
n137	HIV-1 specific peak	26.7	1
n138	Primer -dimer plus HIV-1 specific peak	23.61	
n139	HIV-1 specific peak	25.27	1
n140	HIV-1 specific peak	26.63	1
n141	Primer-dimer	25.12	
n142	Primer -dimer plus HIV-1 specific peak	23.5	
n143	Primer -dimer plus HIV-1 specific peak	27	
n144	HIV-1 specific peak	26.5	1
n145	Primer-dimer	25.57	
n146	Primer -dimer plus HIV-1 specific peak	23.3	
n147	HIV-1 specific peak	26.2	1
n148	No amplification	undet	
n149	No amplification	undet	
n150	Primer-dimer	25.41	

PCR was performed with DNA from 150 HIV-1 negative blood donors as described in Materials and Methods.
Copy number was quantified by extrapolation of the Ct value from the standard curve created automatically by the Applied Biosystems 7500 Real-Time PCR instrument. Copy number <1 refers to values in the range 0.1 - 0.5.
A, B, C, D refer to the dissociation curve in Figure 2, panel A.
Results are summarized in the table of Figure 2, panel A.
