# Supplementary material for: A Real Time PCR Platform for the Simultaneous Quantification of Total and Extrachromosomal HIV DNA Forms in Blood of HIV-1 Infected Patients
Source: PLoS One. 2014 Nov 3;9(11):e111919. doi: 10.1371/journal.pone.0111919 (PMC4218859; doi:10.1371/journal.pone.0111919)
Supplement: Table S2 — pPBS standard curve in assence or presence of HIV-1 negative human DNA. (PDF) [file pone.0111919.s004.pdf]

Table S2 pPBS standard curve in assence or presence of HIV-1 negative human DNA

Copy number	minus BK DNA		plus BK DNA		n
	Ct mean	SD	Ct mean	SD	
1000	16.46	0.07	16.67	0.12	8
300	18.22	0.09	18.44	0.04	8
100	19.87	0.20	20.08	0.01	8
30	21.64	0.32	21.84	0.49	8
10	23.23	0.51	23.29	1.01	8
2	25.35	0.59	25.47	0.89	8
	y=-3.295x+26.444		y=-3.260x+26.509		
