# Supplementary material for: A Real Time PCR Platform for the Simultaneous Quantification of Total and Extrachromosomal HIV DNA Forms in Blood of HIV-1 Infected Patients
Source: PLoS One. 2014 Nov 3;9(11):e111919. doi: 10.1371/journal.pone.0111919 (PMC4218859; doi:10.1371/journal.pone.0111919)
Supplement: Table S3 — Ct value and copy number. (PDF) [file pone.0111919.s005.pdf]

Table S3 Ct value and copy number

Ct	Qty	Copy no.
16.55	1.00E+03	1016
16.53	1.00E+03	1026
16.38	1.00E+03	1145
16.51	1.00E+03	1042
16.41	1.00E+03	1117
16.39	1.00E+03	1134
16.49	1.00E+03	1056
16.39	1.00E+03	1134
18.39	300	280
18.15	300	331
18.23	300	313
18.23	300	312
18.26	300	307
18.11	300	341
18.26	300	307
18.15	300	330
20.03	100	88
19.52	100	127
19.74	100	109
20.10	100	85
19.92	100	96
19.71	100	111
20.06	100	87
19.88	100	99
22.03	30	22
21.93	30	23
21.36	30	35
21.36	30	35
21.32	30	36
21.30	30	37
21.79	30	26
21.29	30	37
21.56	30	30
21.66	30	28
22.21	30	19
21.42	30	34
21.29	30	37
21.41	30	34
22.01	30	22
21.46	30	33
21.85	30	25
21.48	30	32
21.35	30	35
22.10	30	21
21.49	30	32
22.09	30	21
21.56	30	30
22.12	30	21
22.58	10	15
23.17	10	10
22.54	10	15
23.25	10	9
23.12	10	10
23.17	10	10
22.76	10	13
22.91	10	12
23.92	10	6

23.24	10	9
24.33	10	4
23.27	10	9
23.72	10	7
24.03	10	5
23.20	10	10
24.21	10	5
23.40	10	8
22.53	10	15
22.99	10	11
22.79	10	13
22.69	10	14
23.16	10	10
23.21	10	10
26.21	2	1
24.81	2	3
24.71	2	3
24.66	2	3
25.37	2	2
24.60	2	4
26.27	2	1
24.63	2	4
25.37	2	2
25.32	2	2
25.56	2	2
25.38	2	2
25.43	2	2
24.36	2	4
25.46	2	2
24.84	2	3
25.83	2	2
25.76	2	2
25.89	2	1
25.44	2	2
26.42	2	1

These Ct derived from 8 indipendent or more PCR experiments.
The resulting mean standard curve Y=-3.295x+26.444 was used to estrapolate the copy number from the Ct value.
The data are summarized in Table S4.
