# Supplementary material for: A Real Time PCR Platform for the Simultaneous Quantification of Total and Extrachromosomal HIV DNA Forms in Blood of HIV-1 Infected Patients
Source: PLoS One. 2014 Nov 3;9(11):e111919. doi: 10.1371/journal.pone.0111919 (PMC4218859; doi:10.1371/journal.pone.0111919)
Supplement: Table S4 — Method reproducibility calculated by the percentage of the coefficient of variation for Ct value (A) and for copy number (B). (PDF) [file pone.0111919.s006.pdf]

Table S4 Method reproducibility calculated by the percentage of the coefficient of variation for Ct value (A) and for copy number (B)

(A)

Copy number	Ct mean	SD	$\%CV_{Ct}$	95% CI	n
1000	16.46	0.07	0.43	16.4-16.52	8
300	18.22	0.09	0.48	18.15-18.29	8
100	19.87	0.20	1.01	19.7-20.04	8
30	21.64	0.32	1.46	21.51-21.78	24
10	23.23	0.51	2.22	23-23.45	23
2	25.35	0.59	2.33	25.08-25.62	21
Mean %CVCt			*1.32*		

(B)

Copy number	Copy no. mean	SD	$\%CV_{Cn}$	95% CI	n
1000	1084	54	4.96	1039-1129	8
300	315	19	6.03	299-331	8
100	100	15	14.50	88-112	8
30	29	6	20.84	27-32	24
10	10	3	32.28	9-11	23
2	2	1	40.39	2-3	21
Mean %CVCn			*20*		
