# Supplementary material for: A Real Time PCR Platform for the Simultaneous Quantification of Total and Extrachromosomal HIV DNA Forms in Blood of HIV-1 Infected Patients
Source: PLoS One. 2014 Nov 3;9(11):e111919. doi: 10.1371/journal.pone.0111919 (PMC4218859; doi:10.1371/journal.pone.0111919)
Supplement: Table S5 — Quantification of total HIV DNA copy number in three different samples tested in three separate experiments. (PDF) [file pone.0111919.s007.pdf]

Table S5 Quantification of total HIV DNA copy number in three different samples tested in three separate experiments

	Sample 1			Sample 2			Sample 3		
Run	Exp 1	Exp 2	Exp 3	Exp 1	Exp 2	Exp 3	Exp 1	Exp 2	Exp 3
1st qPCR	6	6	5	30	33	33	6	9	11
1st qPCR	3	8	5	32	37	32	8	6	10
2nd qPCR	3	6	6	38	37	34	8	8	5
2nd qPCR	4	4	1	38	33	36	5	8	8
2nd qPCR	2	7	6	32	36	42	8	6	7
2nd qPCR	4	3	4	31	36	39	11	7	10
2nd qPCR	3	3	5	39	32	40	10	9	5
2nd qPCR	2	6	4	34	35	33	5	8	8
Coefficient of variation	38.59%	34.36%	35.63%	10.44%	5.62%	10.40%	28.85%	15.58%	28.35%
Lower 95% CI of mean	2	4	3	31	33	33	6	7	6
Upper 95% CI of mean	4	7	6	37	37	39	9	9	10
Sum	27	43	36	274	279	289	61	61	64
Copies/µg of DNA	7	11	9	68	70	72	15	15	16

The result was obtained dividing the sum of the copy number from a total of eight replicates (two 0.5 µg replicates in the 1st qPCR and six 0.5 µg replicates in the 2nd qPCR) by 4 and expressed as HIV DNA copy number/µg of DNA. See details in Materials and Methods section for samples quantified near or detected below the QL (<2 copies) in the 1st qPCR.
