# Supplementary material for: A Real Time PCR Platform for the Simultaneous Quantification of Total and Extrachromosomal HIV DNA Forms in Blood of HIV-1 Infected Patients
Source: PLoS One. 2014 Nov 3;9(11):e111919. doi: 10.1371/journal.pone.0111919 (PMC4218859; doi:10.1371/journal.pone.0111919)
Supplement: Table S6 — Cross-contamination level of HMW DNA measured in eluate fraction by qPCR of β-actin housekeeping gene. (PDF) [file pone.0111919.s008.pdf]

Table S6 Cross-contamination level of HMW DNA measured in eluate fraction by qPCR of β-actin housekeeping gene

Sample	ng/qPCR	ng in eluate fraction	% of contamination
1	3.4	180.2	5.1
2	2.7	148.5	4.2
3	1.7	90.1	2.6
4	3.5	185.5	5.3
5	2.2	121.0	3.4
6	3.2	169.6	4.8
7	3.5	185.5	5.3
8	2.3	115.0	3.3
9	3.5	201.25	5.7
10	4.3	227.9	6.5
11	3.0	172.5	4.9
12	2.9	145.0	4.1
13	2.9	153.7	4.4
14	2.4	127.2	3.6
15	2.6	135.2	3.8
16	4.8	254.4	7.2
17	4.5	238.5	6.8
18	4.0	212.0	6.0
19	4.7	230.3	6.5
20	4.5	238.5	6.8
Mean±SD	3.5±1.2	176.6±47.1	5.0±1.4

The contamination from HMW DNA fragments was evaluated in PCR reaction (ng/qPCR, 2 µl of eluate fraction were tested in triplicate) and in the total volume of the eluate fraction (varying from sample to sample). Percentage of contamination is expressed as % versus the total DNA amount loaded in the chromatographic column (3529 ng, see Materials and Methods).
