# Supplementary material for: A Real Time PCR Platform for the Simultaneous Quantification of Total and Extrachromosomal HIV DNA Forms in Blood of HIV-1 Infected Patients
Source: PLoS One. 2014 Nov 3;9(11):e111919. doi: 10.1371/journal.pone.0111919 (PMC4218859; doi:10.1371/journal.pone.0111919)
Supplement: Table S7 — Recovery test of extrachromosomal forms. (PDF) [file pone.0111919.s009.pdf]

Table S7 Recovery test of extrachromosomal forms

	Copy number of exogenous pEXg standard					
	10^2 *			10^3 *		
	Copy no. /qPCR	Expected copy no.	Recovery (%)**	Copy no. /qPCR	Expected copy no.	Recovery (%)**
	9	10	89	92	100	92
Sample 1	10	10	98	90	100	90
	8	10	80	103	100	103
	9	10	92	95	100	95
Sample 2	8	10	85	90	100	90
	10	10	99	99	100	99
	12	10	120	97	100	97
Sample 3	8	10	84	100	100	100
	10	10	100	95	100	95
	10	10	100	82	100	82
Sample 4	8	10	85	100	100	100
	10	10	100	110	100	110
Mean± SD	9±1		94±11	96±7		96±7

* 10^2 or 10^3 copies of pEXg were added to 5 µg of cellular DNA at the beginning of column separation procedure.

** Recovery of the pEXg was calculated as the observed/expected copy number.
