# Supplementary material for: A Real Time PCR Platform for the Simultaneous Quantification of Total and Extrachromosomal HIV DNA Forms in Blood of HIV-1 Infected Patients
Source: PLoS One. 2014 Nov 3;9(11):e111919. doi: 10.1371/journal.pone.0111919 (PMC4218859; doi:10.1371/journal.pone.0111919)
Supplement: Table S8 — Characteristics of total, unintegrated and integrated HIV DNA measurements in samples. (PDF) [file pone.0111919.s010.pdf]

Table S8 Characteristics of total, unintegrated and integrated HIV DNA measurements in samples

		n	Sample quantified	(%)	Sample detected*	(%)
**Total HIV DNA**						
	Total (n, 195)	195	161	83	34	17
	With plasma viremia <50 copies/ml (n, 123)	123	103	84	20	16
	With plasma viremia >50 copies/ml (n, 72)	72	58	81	14	19
	With CD4+ T cell count <350 cells/µl (n,96)	96	91	95	5	5
	With CD4+ T cell count >350 cells/µl (n, 99)	99	82	83	17	17
	ART-naïve (n, 32)	32	26	81	6	19
	ART-experienced (n, 163)	163	135	83	28	17
	Mean±SD			84±5		16±5
**Unintegrated HIV DNA**		195				
	Total	123	139	71	56	29
	With plasma viremia <50 copies/ml	72	85	69	38	31
	With plasma viremia >50 copies/ml	96	54	75	18	25
	With CD4+ T cell count <350 cells/µl	99	69	72	27	28
	With CD4+ T cell count >350 cells/µl	32	70	71	29	29
	ART-naïve	163	24	75	8	25
	ART-experienced		115	71	48	29
	Mean±SD			72±2		28±2
**Integrated HIV DNA**						
	Total	195	134	69	61	31
	With plasma viremia <50 copies/ml	123	82	67	41	33
	With plasma viremia >50 copies/ml	72	52	72	20	28
	With CD4+ T cell count <350 cells/µl	96	67	70	29	30
	With CD4+ T cell count >350 cells/µl	99	67	68	32	32
	ART-naïve	32	23	72	9	28
	ART-experienced	163	111	68	52	32
	Mean±SD			69±2		31±2

* Samples with positive PCR amplification, below the limit of quantification (2 copies).
