# Supplementary material for: A Real Time PCR Platform for the Simultaneous Quantification of Total and Extrachromosomal HIV DNA Forms in Blood of HIV-1 Infected Patients
Source: PLoS One. 2014 Nov 3;9(11):e111919. doi: 10.1371/journal.pone.0111919 (PMC4218859; doi:10.1371/journal.pone.0111919)
Supplement: Table S9 — Correlations between study parameters in blood samples. (PDF) [file pone.0111919.s011.pdf]

Table S9 Correlations between study parameters in blood samples

Sample	HIV-1 RNA versus				CD4+ T cell count versus				HIV-1 RNA vs CD4+ T cell count
	Total HIV DNA normalized to		Unintegrated HIV DNA normalized to		Total HIV DNA normalized to		Unintegrated HIV DNA normalized to		
	µg DNA	10^4 CD4+	µg DNA	10^4 CD4+	µg DNA	10^4 CD4+	µg DNA	10^4 CD4+	
All (n=195)	0.21[a] (<0.005)[b]	0.25 (<0.005)	0.26 (<0.005)	0.31 (<0.0001)	-0.03 (0.68)	-0.48 (<0.0001)	-0.09 (0.23)	-0.52 (<0.0001)	-0.14 (0.05)
MDR [c] (n=85)	0.13 (0.24)	0.22 (0.04)	0.14 (0.21)	0.27 (0.01)	-0.003 (0.98)	-0.40 (<0.005)	-0.05 (0.67)	-0.43 (<0.0001)	-0.25 (0.02)
non-MDR (n=110)	0.28 (<0.005)	0.31 (<0.005)	0.39 (<0.0001)	0.37 (<0.0001)	-0.07 (0.46)	-0.53 (<0.0001)	-0.13 (0.18)	-0.58 (<0.0001)	-0.11 (0.25)
Naïve (n=32)	0.42 (0.02)	0.66 (<0.0001)	0.64 (<0.0001)	0.68 (<0.0001)	-0.38 (0.03)	-0.75 (<0.0001)	-0.55 (<0.005)	-0.77 (<0.0001)	-0.57 (<0.005)
ART (n=163)[d]	0.15 (0.06)	0.24 (<0.005)	0.17 (0.03)	0.32 (<0.0001)	0.06 (0.46)	-0.40 (<0.0001)	0.005 (0.95)	-0.47 (<0.0001)	-0.26 (<0.005)
Under RAL (n=90)[e]	0.14 (0.20)	0.17 (0.12)	0.13 (0.22)	0.23 (0.03)	-0.02 (0.83)	-0.41 (<0.0001)	-0.005 (0.96)	-0.40 (<0.0001)	-0.19 (0.08)
HIV-1 RNA >50 cp/ml(n=72)	-0.04 (0.75)	0.40 (0.005)	0.10 (0.42)	0.49 (<0.0001)	-0.004 (0.98)	-0.55 (<0.0001)	-0.12 (0.33)	-0.60 (<0.0001)	-0.18 (0.14)

[a] Sperman's correlation coefficient and [b] p-value are reported in the table.

[c] Samples collected from patients with multidrug-resistant HIV-1 infection.

[d] 2NRTI,PI (n=47, 29%); 2NRTI,PI,II (n=44, 27%); 2NRTI,NNRTI (n=26, 16%); 2NRTI,NNRTI,II (n=13, 8%); 2NRTI,II,CCR5I (n=6, 4%); NRTI,PI,II (n=6, 4%); NRTI,NNRTI,II,CCR5I (n=6, 4%); NRTI,PI,II,CCR5I (n=5, 3%); 2NRTI,NNRTI,II,CCR5I (n=4, 2%); NNRTI,II,CCR5I (n=3, 2%); NRTI,II (n=2, 1%); 2NRT,II (n=1, 1%).

[e] 2NRTI,PI,II (n=44, 49%); 2NRTI,NNRTI,II (n=13, 14%); 2NRTI,II,CCR5I (n=6, 7%); NRTI,PI,II (n=6, 7%); NRTI,NNRTI,II,CCR5I (n=6, 7%); NRTI,PI,II,CCR5I (n=5, 6%); 2NRTI,NNRTI,II,CCR5I (n=4, 4%); NNRTI,II,CCR5I, (n=3, 3%); NRTI,II (n=2, 2%); 2NRT,II (n=1, 1%).
