# Supplementary material for: A Real Time PCR Platform for the Simultaneous Quantification of Total and Extrachromosomal HIV DNA Forms in Blood of HIV-1 Infected Patients
Source: PLoS One. 2014 Nov 3;9(11):e111919. doi: 10.1371/journal.pone.0111919 (PMC4218859; doi:10.1371/journal.pone.0111919)
Supplement: Table 10 — Correlation between study parameters in patients at the beginning and the end of the observation period. (PDF) [file pone.0111919.s012.pdf]

Table S10 Correlation between study parameters in patients at the beginning and the end of the observation period.

Patients		HIV-1 RNA versus				CD4+ T cell count versus				HIV-1 RNA vs CD4+ T cell count
		Total HIV DNA normalized to		Unintegrated HIV DNA normalized to		Total HIV DNA normalized to		Unintegrated HIV DNA normalized to		
		µg DNA	10^4 CD4+	µg DNA	10^4 CD4+	µg DNA	10^4 CD4+	µg DNA	10^4 CD4+	
	START	0.24	0.40	0.36	0.48	-0.11	-0.67	-0.22	-0.70	-0.29
	p	*0.11*	*0.01*	*0.01*	*0.001*	*0.47*	*<0.0001*	*0.14*	*<0.0001*	*0.06*
	STOP	0.10	0.03	0.21	0.24	-0.05	-0.53	0.10	-0.53	0.00
All (n=45)	*p*	*0.51*	*0.85*	*0.17*	*0.11*	*0.73*	*<0.005*	*0.50*	*<0.005*	*0.98*
	START	0.35	0.54	0.54	0.56	0.60	0.46	0.46	0.00	-0.35
	p	*0.52*	*0.35*	*0.35*	*0.35*	*0.35*	*0.45*	*0.45*	-	*0.52*
	STOP	0.54	0.71	0.54	0.71	0.41	0.30	0.41	0.10	-0.35
Naïve (n=5)	*p*	*0.35*	*0.23*	*0.35*	*0.23*	*0.52*	*0.68*	*0.52*	*0.95*	*0.52*
	START	0.14	0.40	0.30	0.45	-0.30	-0.62	-0.44	-0.73	-0.53
	p	*0.58*	*0.10*	*0.23*	*0.06*	*0.23*	*0.01*	*0.07*	*<0.005*	*0.02*
	STOP	0.19	0.20	0.24	0.22	0.14	-0.12	0.13	-0.42	-0.20
ART (n=18)[a]	*p*	*0.46*	*0.42*	*0.34*	*0.37*	*0.57*	*0.64*	*0.60*	*0.09*	*0.42*
	START	0.29	0.61	0.44	0.65	0.33	-0.57	0.04	-0.63	-0.30
	p	*0.27*	*0.01*	*0.09*	*0.01*	*0.21*	*0.02*	*0.89*	*0.01*	*0.25*
	STOP	0.00	0.21	0.06	0.50	-0.24	-0.75	-0.07	-0.88	-0.53
Under RAL (n=16)[b]	*p*	*1.00*	*0.45*	*0.81*	*0.05*	*0.37*	*<0.005*	*0.79*	*<0.0001*	*0.03*
	START	0.34	0.62	0.47	0.69	-0.10	-0.68	-0.21	-0.70	-0.50
	p	*0.05*	*<0.0001*	*<0.005*	*<0.0001*	*0.57*	*<0.0001*	*0.24*	*<0.0001*	*<0.005*
	STOP	0.12	0.22	0.17	0.38	0.00	-0.53	0.10	-0.65	-0.31
ART & Under RAL (n=34)	*p*	*0.51*	*0.20*	*0.35*	*0.02*	*0.99*	*<0.005*	*0.57*	*<0.0001*	*0.07*

[a] 2NRTI,PI (n=13, 72%) and 2NRTI,NNRTI (n=5, 28%).

[b] 2NRTI,PI,II (n=10, 63%); 2NRTI,NNRTI,II (n=2, 13%); 2NRTI,II (n=1, 6%); NRTI,II (n=1, 6%); 2NRTI,CCR5I,II (n=1, 6%); NRTI,PI,CCR5I,II (n=1, 6%).
